# EBV abortive lytic cycle promotes nasopharyngeal carcinoma progression through recruiting monocytes and regulating their directed differentiation

**DOI:** 10.1371/journal.ppat.1011934

**Published:** 2024-01-11

**Authors:** Xiaoting Xu, Nannan Zhu, Junming Zheng, Yingying Peng, Mu-Sheng Zeng, Kai Deng, Chaohui Duan, Yan Yuan

**Affiliations:** 1 Laboratory of Clinical, Sun Yat-sen Memorial Hospital of Sun Yat-sen University, Guangzhou, China; 2 Institute of Human Virology, Zhongshan School of Medicine, Sun Yat-Sen University, Guangzhou, Guangdong, China; 3 State Key Laboratory of Oncology in South China, Sun Yat-sen University Cancer Center, Guangzhou, China; 4 Guanghua School of Stomatology, Hospital of Stomatology, Sun Yat-sen University, Guangdong Provincial Key Laboratory of Stomatology, Guangzhou, China; 5 Institute for Advanced Medical Research, Shandong University, Jinan, China; Brigham and Women’s Hospital, UNITED STATES

## Abstract

Epstein-Barr virus (EBV) is associated with several types of human cancer including nasopharyngeal carcinoma (NPC). The activation of EBV to the lytic cycle has been observed in advanced NPC and is believed to contribute to late-stage NPC development. However, how EBV lytic cycle promotes NPC progression remains elusive. Analysis of clinical NPC samples indicated that EBV reactivation and immunosuppression were found in advanced NPC samples, as well as abnormal angiogenesis and invasiveness. To investigate the role of the EBV lytic cycle in tumor development, we established a system that consists of two NPC cell lines, respectively, in EBV abortive lytic cycle and latency. In a comparative analysis using this system, we found that the NPC cell line in EBV abortive lytic cycle exhibited the superior chemotactic capacity to recruit monocytes and polarized their differentiation toward tumor-associated macrophage (TAM)-like phenotype and away from DCs, compared to EBV-negative or EBV-latency NPC cells. EBV-encoded transcription activator ZTA is responsible for regulating monocyte chemotaxis and TAM phenotype by up-regulating the expression of GM-CSF, IL-8, and GRO-α. As a result, TAM induced by EBV abortive lytic cycle promotes NPC angiogenesis, invasion, and migration. Overall, this study elucidated the role of the EBV lytic life cycle in the late development of NPC and revealed a mechanism underlying the ZTA-mediated establishment of the tumor microenvironment (TME) that promotes NPC late-stage progression.

## Author summary

Recently increasing evidence suggests that the lytic life cycle of certain oncoviruses contributes to tumorigenesis. This is in contrary to traditional wisdom that virally associated tumors are mainly attributed to the latent cycle of a tumor virus but not to the lytic phase simply because a lytic virus in general leads to lysis and death of host cells. However, the paradoxical roles of viral lytic cycle in tumorigenesis are still elusive. In the current study, we investigated the role of the EBV lytic cycle in NPC development. With a breakthrough in methodology that allows us to establish an EBV abortive lytic phase cell model, we compared the NPC cells in latent and abortive lytic phase respectively and found that the NPC cells in EBV abortive lytic cycle exhibited the superior chemotactic capacity to recruit monocytes and polarized their differentiation toward TAM-like phenotype and away from DCs. As a consequence, TAM induced by EBV abortive lytic cycle promotes NPC angiogenesis, invasion, and migration. Our study revealed the role of EBV lytic life cycle in NPC development that regulates monocytes to create a tumor-promoting microenvironment and possible implications for novel therapeutic strategy to treat NPC late-stage patients.

## Introduction

Epstein-Barr virus (EBV) is a ubiquitous human virus infecting 90% of the global population. It is the first human tumor virus discovered and is known to be associated with several types of human cancer, including Burkitt’s lymphoma (BL), Hodgkin’s lymphoma (HL), nasopharyngeal carcinoma (NPC), and a subset of gastric carcinoma (GC) [1]. Nasopharyngeal carcinoma is an endemic disease with high incidence in Southern China [2].

The life cycle of EBV consists of lytic and latent phases [3]. EBV infection in NPC cells is mainly latent. Examination of viral gene expression in NPC cells infected with EBV revealed representative type II latent EBV infection, which expresses EBNA1, LMP1, LMP2A, two small RNAs, and BART transcripts. The roles of these latent genes in the carcinogenic process have been intensively studied for decades [4,5]. Although EBV lytic cycle has long been assumed not to contribute to oncogenesis simply because the lytic cycle, in general, leads to lysis and death of host cells, nowadays the lytic cycle and some viral lytic proteins have also been implicated to be crucial for tumor development and progression [3]. An EBV transcriptome study revealed that EBV lytic genes are co-expressed with cellular cancer-associated pathways [6]. The EBV lytic cycle was found to exist in NPC biopsies [7]. The paradoxical roles of EBV lytic cycle and lytic proteins in tumorigenesis are still elusive, but one possibility is that some lytic proteins are expressed outside of the traditional lytic cycle, which allows specific lytic genes to be expressed in the absence of fully lytic replication leading to virion production. This is referred to as abortive lytic cycle [8,9]. This notion was supported by observations that EBV lytic genes, often immediate-early and early genes, were detected in a small percentage of NPC cells, but lack late genes, especially those encoding late structural proteins, thus failing to produce new virions or cause cell lysis. EBV also has a short pre-latent abortion lytic cycle immediately after infecting cells [10]. The biological significance of abortive lytic cycle in EBV-associated cancers remains to be explored.

Another common characteristic of EBV-associated NPC is the heavy infiltration of leukocytes, especially in advance-staged NPC [11]. The coexistence of tumor-infiltrating leukocytes with EBV-infected NPC cells represents a distinct tumor microenvironment (TME), which renders tumor immune evasion and promotes NPC progression [11]. Studies showed that EBV alters host cell signaling to facilitate tumor development intracellularly [12], and EBV-infected cancer cells communicate with stromal cells through the secretion of cytokines and chemokines intercellularly to repress immune surveillance and enhance metastasis [13]. EBV infection offers nasopharyngeal carcinoma cells a growth advantage in TME *in vivo* [14]. In TME, tumor immunity initiation involves capturing and processing tumor antigens by professional antigen-presenting cells (APCs) to exert cytotoxic anti-tumor effects [15]. APCs in solid tumors are mainly dendritic cells (DC) and macrophages (Mφ), which are derived from circulating blood monocytes migrating to tissues and differentiating into resident tissue monocyte-derived macrophages [16] and monocyte-derived DCs [17]. As specialized antigen-presenting cells, dendritic cells can specifically activate T cells and initiate anti-tumor immunity. In nasopharyngeal carcinoma, mature DC is positively correlated with the abundance of lymphocyte infiltration [18]. Macrophages can further differentiate into pro-inflammatory type M1 or anti-inflammatory type M2, and the latter are considered tumor-associated macrophages (TAMs) in TME. TAMs exhibit the capacity to promote cancer cell proliferation, immunosuppression, and angiogenesis to support tumor growth and metastasis [19]. The abundance of TAMs in tumors is often associated with poor prognosis. In solid tumors, TME promotes monocytes preferentially generating immunosuppressive TAMs and inhibiting DCs, which promote tumor progression and immune escape [16,20]. Still, the molecular basis of this in NPC is largely unknown.

Distant metastasis of nasopharyngeal carcinoma with angiogenesis is one of the primary causes of death of NPC, and metastasis occurred in three-quarters of patients [21]. It is believed that tumor angiogenesis causes tumor cells to enter the blood circulation and plays an essential role in tumor progression and metastasis [22]. Therefore, elucidation of the mechanism underlying the angiogenesis and metastasis of nasopharyngeal carcinoma is crucial. In the current study, we attempted to investigate the contribution of EBV to forming a unique TME, which has been postulated to play an essential role in supporting the growth and progression of NPC cells in patients. We found that abortive lytic cycle of EBV can specifically recruit monocytes and direct their differentiation to TAMs, thus promoting angiogenesis and metastasis of NPC.

## Results

### EBV Lytic gene expression is associated with advanced NPC and immunosuppression

**Fig 1:**
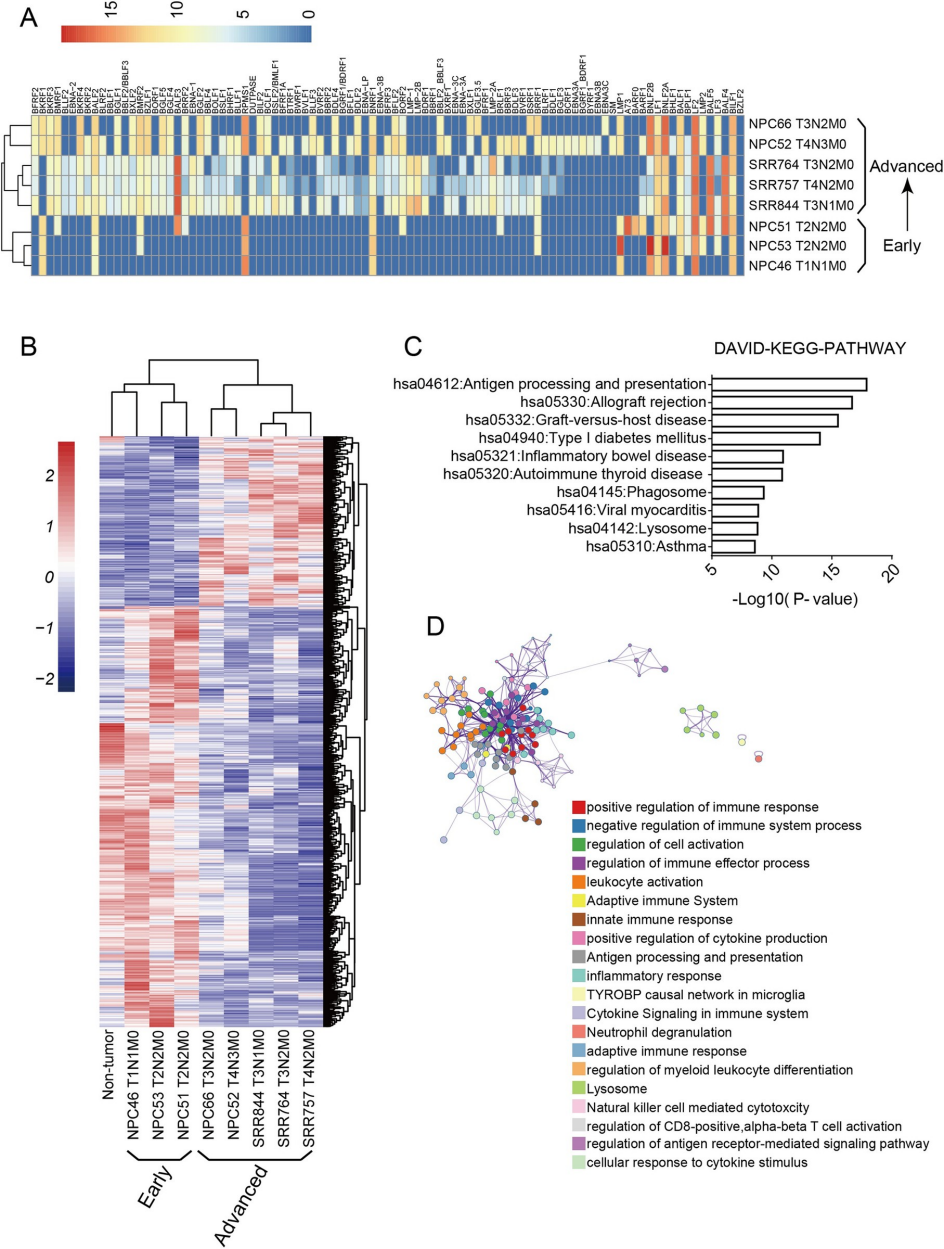
EBV lytic gene expression is associated with advanced NPC and immunosuppression. **(A)** Heatmap illustrating relative EBV gene expression profiles in NPC samples from different TNM stages. Unsupervised clustering of genes (x-axis) and nasopharyngeal carcinoma samples (y-axis) was performed by complete-linkage clustering. **(B)** Heatmap showing genes differentially expressed between the early-stage NPCs samples and the late-stage NPCs samples. RNA-seq reads were aligned to hg19/GRCh37 human reference genome and visualized on standard scaling. **(C)**The down-regulated genes in advanced NPC were analyzed by DAVID-KEGG. Top 10 enriched signaling pathways were displayed (the abscissa is -log10 (P-value); the ordinate is pathway name). **(D)** Cluster network diagram of the genes that are down-regulated in advanced NPC via Metascape. The darkness of colors represents the enrichment into the pathway or biological process.

Recent transcriptome sequencing analysis revealed that in some NPC cells, the expression of the EBV genome is not restricted to type II latency but also expresses type III latency and lytic genes [23]. The significance of the expression of lytic genes in NPC pathogenesis remains unknown. To approach this question, we analyzed eight NPC clinical samples diagnosed in different TNM (tumor-node-metastasis) stages for their transcription profiles. NPC46, 51, 53 were in the T1 or T2 stages, considering to be early-staged tumors, while SRR844, 757, 764, NPC52, and 66 were in T3 or T4 stages, considering late-staged tumors. When RNA-Seq reads obtained from these NPC tumors were aligned to the EBV genome (GenBank: AJ507799.2) and visualized by a heatmap of EBV transcript RPKMs (Reads Per Kilobase per Million mapped reads) across the viral genome, it was revealed that in early stage NPC samples, EBV was mainly in latent phase, while in the advanced stage NPC samples, EBV lytic genes were highly expressed, suggesting that EBV gene expression was switched from latent to lytic phase along with the development of NPC from early to late stages (Fig 1A). This finding is consistent with a previous clinical statistical study that plasma EBV-DNA copy number of NPC patients is positively correlated with TNM stages [24].

The biological significance of the various EBV expression profiles to NPC cells was explored by analyzing the host transcriptome changes corresponding to the EBV gene expression patterns and NPC stages. The properly filtered and normalized RNA-Seq reads of these NPC samples were aligned to the human genome (hg19/GRCh37). The RPKM of each gene in the early-stage NPCs (NPC46, NPC51, and NPC53) and the late-stage NPCs (SRR844, SRR764, NPC66, SRR757, and NPC52) were analyzed by a two-tailed T-test for differentially expressed genes (DEGs). This analysis identified 1721 differentially expressed genes of early versus late NPCs. These genes were visualized on standard scaling, of which 1229 genes were down-regulated in the advanced NPC samples, and 492 genes were up-regulated in the advanced NPCs compared to the early NPCs (Fig 1B). The 1229 down-regulated DEGs in advanced NPCs were subjected to DAVID KEGG pathway analysis and METASCAPE Gene enrichment analysis to identify enriched gene sets and signaling pathways. Interestingly among the top-ranked enrichment list of altered pathways between the early and late NPCs are the terms of regulation of immune systems, with the antigen presentation related signaling pathway ranked number one in the list. The result indicates that the antigen presentation signaling pathway is down-regulated or inactivated in the late-staged NPC (Fig 1C and 1D).

### NPC cells with EBV abortive lytic cycle exhibit enhancive chemotaxis capacity to recruit monocytes

**Fig 2:**
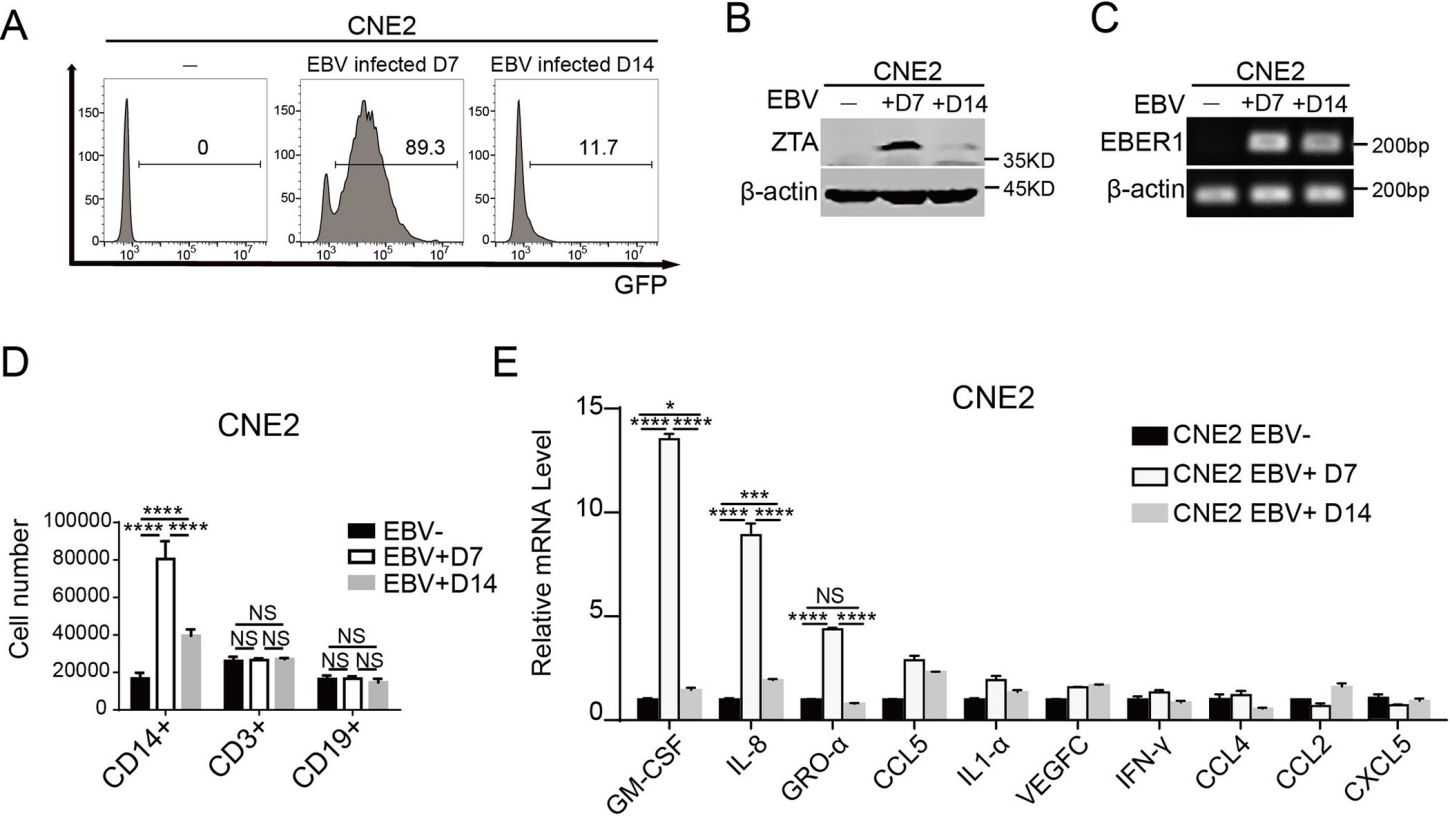
CNE2 cells in EBV abortive lytic phase exhibit enhanced capacity of monocytes chemotaxis. **(A)** The switch of EBV phase in EBV^+^CNE2-Day7 and -Day14 was presented by GFP flow cytometry. **(B)** ZTA expression levels in EBV^+^CNE2-Day7 and -Day14 were analyzed by Western blotting. **(C)** EBER1 expression levels were quantified by Reverse-transcriptional PCR. **(D)** The number of immune cells recruited by CNE2 in different EBV phase (n = 4). **(E)** The trend of cytokine mRNA level change was detected by real-time PCR (n = 3). Data are presented as the mean±SEM. * P < 0.*05*, ** P < 0.*01*, *** P < 0.*001*, **** P < 0.*0001*, *NS*, *not significant*.

RNA-Seq transcriptome study demonstrated that EBV lytic or abortive lytic replication occurs in advanced NPCs, and the advanced tumor is associated with significant immunosuppression. Now the question is whether the EBV lytic cycle (or abortive lytic cycle) contributes to the immunosuppression. To address this question, we attempted to establish an *in vitro* model to recapitulate the lytic cycle-associated immunosuppression process in cells. Because of lack of an EBV-positive NPC cell line in stable abortive lytic phase, we utilized two NPC cell lines, that were infected with EBV, but EBV was in transient pre-latent abortive lytic phase and latency phase, respectively. Such a pair of cell lines were generated by co-culturing EBV-negative CNE2 with EBV-positive Akata-BL cells, followed by G418 selecting for 7 days [25,26]. A GFP-tag was inserted in EBV-BXLF1 open reading frame, so it expresses in lytic cycle but no longer expresses after EBV enters latency. On Day 7 after contracting EBV, 89.3% of CNE2 cells expressed high level GFP fluorescein, indicating that the EBV in the cells was in the transient pre-latent abortive lytic cycle. In contrast, on Day 14, only 11.7% of CNE2 expressed weak GFP, indicating that EBV in most of the CNE2 cells had entered latency (Fig 2A). To verify the phases of EBV on Day 7 and Day 14, we examined the expression of ZTA (Z transcription activator) by Western blot (Fig 2B). EBER1 (Epstein-Barr encoded small RNA) is a non-coding small RNA constitutively expressed by EBV and is continuously transcribed throughout the entire latency and lytic cycle of EBV. Reverse-transcriptional PCR (RT- PCR) was used to detect the expression of EBER1 in EBV-infected CNE2 cells to confirm the persistence of EBV (Fig 2C). Western blot and RT-PCR results proved that EBV-infected CNE2 cells on Day 7 was in abortive lytic phase (ZTA^+^/EBER1^+^) and was in latency (ZTA^-^/EBER1^+^) on Day 14. We also infected HK-1 with the same method and observed similar results. HK-1 was in the abortive lytic cycle on the 7th day of EBV infection and entered the latency on the 14th day post-EBV infection (S1A–S1C Fig). The quantification of ZTA and EBER1 expression in CNE2 and HK-1 were performed in S2A and S2B Fig. The full spectrum of EBV gene expression in CNE-EBV+D7 and D14 were presented in heatmap form (S2C Fig).

With the EBV-abortive lytic/latent CNE2 cell system, we set up a Transwell PBMC recruitment assay to explore the effect of EBV lytic cycle on immune cell chemotaxis. Latent EBV^+^CNE2 and abortive lytic EBV^+^CNE2 were seeded in the lower chambers of Transwell, and human peripheral blood mononuclear cells (PBMC) were added into Transwell inserts. After 24 hours, immune cells recruited to the lower chambers were collected and analyzed by flow cytometry to determine the proportion of each type (Fig 2D). The result showed a divergent chemotaxis pattern of immune cells in response to latent and lytic EBV^+^CNE2. The most prominent feature is that CNE2-EBV^+^Day7 cells caused much more CD14^+^ monocytes to chemotactically migrate toward the lower chamber, in contrast to CNE2-EBV^+^Day14 and EBV-negative CNE2 (Fig 2E). Furthermore, in order to completely circumvent the possible influence of EBV-specific T cells, we used THP-1, an immortalized human monocyte cell line, for chemotaxis assay. As shown in S3A Fig, CNE2-D7 clearly promoted THP-1 migration.

The expression of chemokines, inflammatory, and growth factors in abortive lytic EBV^+^CNE2-Day7 and latent EBV^+^CNE2-Day14 was compared using real-time PCR. The result showed that GM-CSF, GRO-α, and IL-8 were the most significantly increased cytokines in CNE2-EBV^+^Day7 compared to CNE2-EBV^+^Day14 (Fig 2F). It is known that these cytokines and chemokines play crucial roles in monocyte differentiation and chemotaxis. GM-CSF (granulocyte-macrophage colony stimulating factor) regulates monocyte differentiation into dendrites [27] or macrophages [28]. On the other hand, IL-8 (interleukin-8, also known as CXCL8) [29] and GRO-α (Human growth Regulation Oncogene α, also known as CXCL1) [30] have chemotactic effects on monocyte-derived cells. Therefore, EBV abortive lytic cycle apparently has a role in promoting the expression of these cytokines, recruiting monocytes, and regulating the differentiation of monocytes. HK-1 on the 7th day of EBV infection also enhanced the ability to recruit monocytes and the expression of monocyte-related cytokines increased significantly (S4A and S4B Fig). Taken together, NPC cells with abortive lytic EBV exhibit chemotactic capability of specific recruitment of monocytes through the expression of particular cytokines.

### NPC cells in EBV abortive lytic cycle regulate monocyte differentiation toward TAM-like phenotype

**Fig 3:**
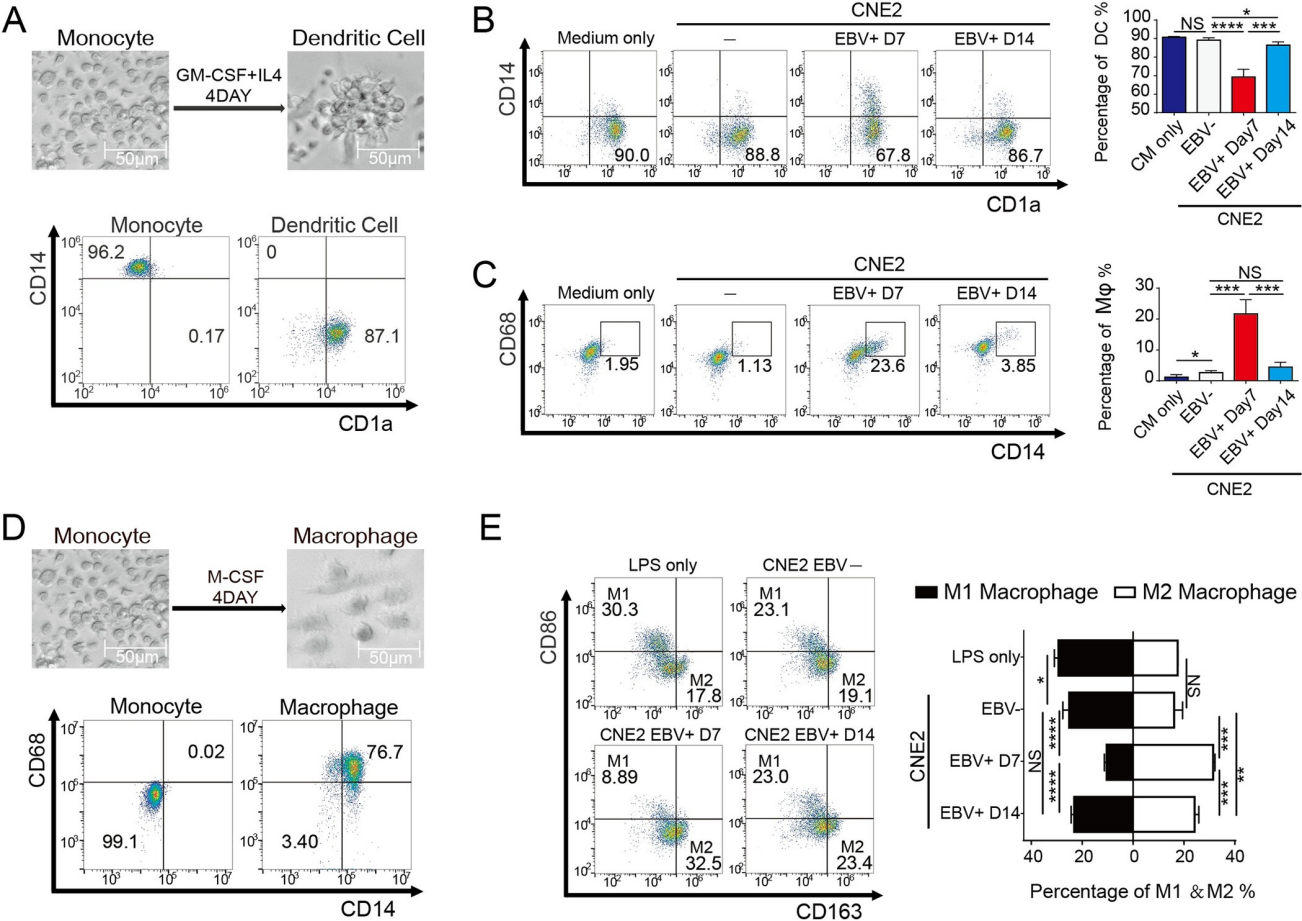
CNE2 cells in EBV abortive lytic phase promote monocyte differentiation toward TAM-like phenotype and away from DCs. **(A)** Changes in cell morphology and cell surface markers during DC differentiation. Scale bar, 50 μm. **(B)** and **(C)** Supernatants of CNE2 with different EBV life cycle were used to treat DCs. The difference in the yield of DCs was examined by CD14&CD1a expression profile. the proportion of DC-d-Ms was examined by CD14&CD68 expression profile (n = 4). **(D)** Changes in cell morphology and cell surface markers during Mφs differentiation. Scale bar, 50 μm. **(E)** Supernatants of CNE2 in different EBV life cycle were used to treat Mφs, the proportion of M1 and M2 subtypes were determined by detecting CD86 and CD163 (n = 4). Data are presented as the mean±SEM. * P < 0.*05*, ** P < 0.*01*, *** P < 0.*001*, **** P < 0.*0001*, *NS*, *not significant*.

Monocytes are myeloid cells circulating in the blood, which can be recruited to inflammatory sites by inflammatory factors and differentiated into macrophages or dendritic cells depending on the microenvironment of the inflammatory site [16]. After showing that the NPC cells with EBV abortive lytic cycle recruit monocytes, we asked if the EBV abortive lytic cycle affects the differentiation of monocytes into DCs or Mφs. To address this question, two systems that allow monocytes to differentiate into DCs and Mφs were established and used to assay the differentiation under the influence of NPCs with the EBV lytic phase. First, monocytes (CD14^+^/CD1a-) isolated from human PBMC were cultured in RPMI 1640 complete medium with GM-CSF and IL-4, which allows monocytes to change from regular and uniform spherical cells to irregular dendritic cell suspension colonies (CD14-/CD1a^+^) [31] (Fig 3A). Then, conditioned media (CM) of latent EBV^+^CNE2 or abortive lytic EBV^+^CNE2 were added to the system, respectively. The influence of these CMs on DC differentiation was determined by monitoring the changes in DCs surface marker expression with flow cytometry. Results showed that the CM of CNE2-EBV^+^Day7 significantly reduced the proportion of DCs in contrast to the CNE2-EBV^-^ CM that had no significant effect on DC differentiation; while the CM of CNE2-EBV^+^ Day14 only had a weak impact on DC differentiation (Fig 3B). In the group treated with the CM of CNE2-EBV^+^Day7, some monocytes recovered high expression of CD14, and the expression of CD68 was up-regulated concurrently, which we considered as DC-derived macrophages (DC-d-Ms) [32] and suggests that EBV lytic cycle increased the ratio of DC-d-Ms (CD14^+^/CD68^+^). In contrast, no such phenomenon was observed in CNE2-EBV^-^ group and CNE2-EBV^+^Day14 group (Fig 3C). In conclusion, CNE2 with abortive lytic EBV can reverse the differentiation of monocytes into DCs, and in turn promote the transformation of some DCs into a macrophage-like phenotype, reducing the yield of DCs.

Second, M-CSF can induce monocytes (CD14^+^/CD68-) differentiating into Mφs (CD14^+^/CD68^+^) [33], which increase in size and appear as irregular pseudopodia (Fig 3D). Macrophages were activated by lipopolysaccharide (LPS) and macrophage subtypes differentiation were induced with the CM of CNE2. After 48 hours, different proportions of M1 and M2 macrophages were obtained. M1 (CD86^+^ CD163-) is a pro-inflammatory phenotype with anti-tumor activity, while M2 (CD86-CD163^+^) is an immunosuppressive cell type and is thought to have TAMs (tumor-associated macrophage) phenotype in solid tumors that can promote tumor progression [34]. We found that CM of CNE2-EBV^+^Day7 significantly reduced the proportion of M1 and, more importantly, induced M2 macrophage differentiation in comparison to CM of CNE2-EBV^-^ and CNE2-EBV^+^Day14 (Fig 3E). Taken together, we verified that the CM of CNE2 with abortive lytic EBV exhibited superior capacity on promoting monocyte differentiation toward TAM-like phenotype and away from DCs. The CM of HK-1-EBV^+^Day7 showed a similar function (S4C-S4E Fig).

### ZTA is the viral mechanism by which EBV abortive lytic cycle regulates monocytes

**Fig 4:**
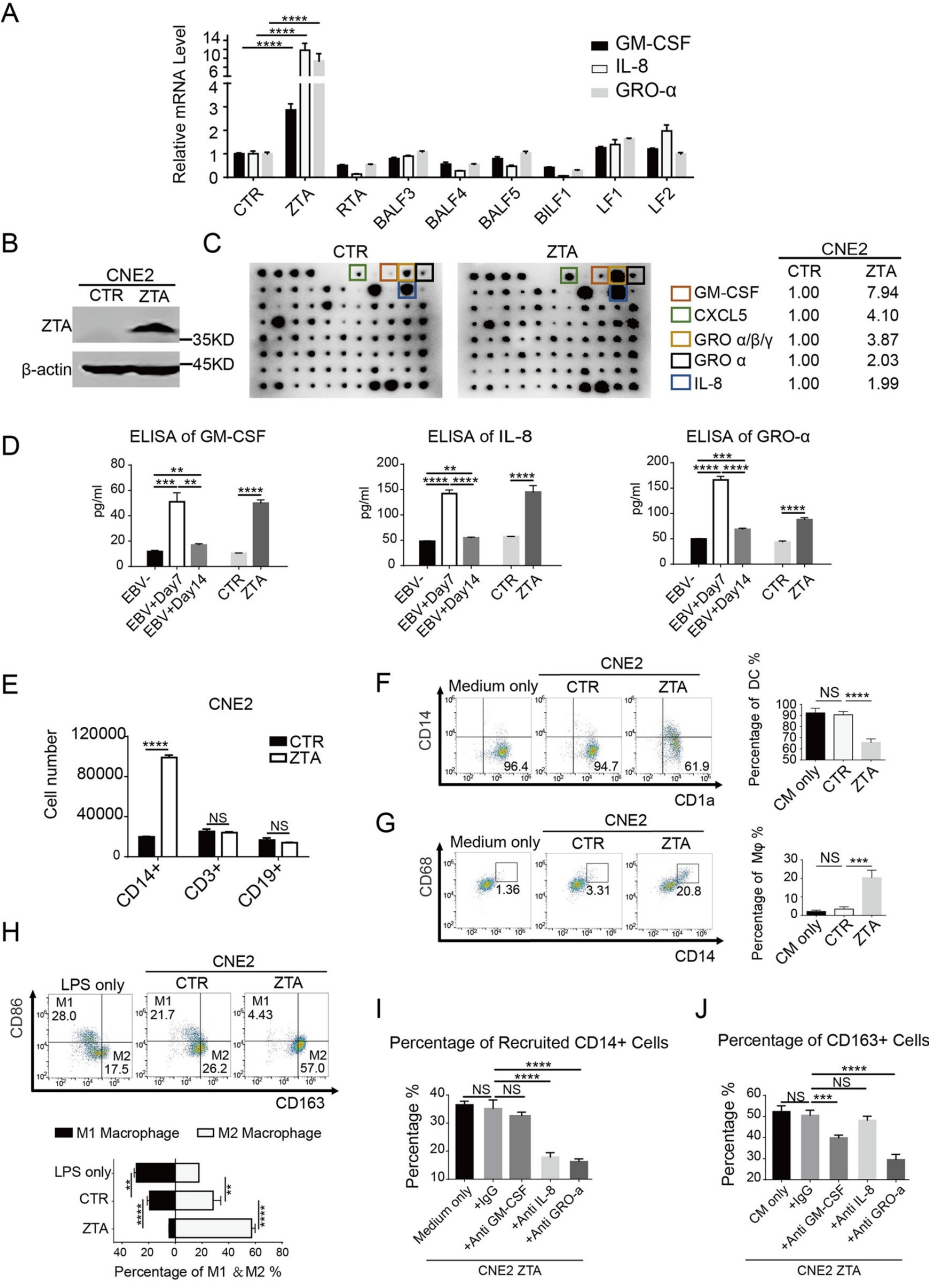
ZTA exhibits the ability to recruit and induce directed differentiation of monocytes. **(A)** The mRNA expression levels of GM-CSF, IL-8 and GRO-α were determined by real-time PCR in CNE2 cells that express the EBV lytic genes as indicated (n = 3). **(B)** Stable expression of ZTA in CNE2 cells confirmed by Western blot. **(C)** Cytokine antibody array identified the differences of cytokine secretion between the supernatants of CNE2-ZTA and CNE2-CTR. The spot signal densities were quantified by Image J. **(D)** The concentrations of GM-CSF, IL-8 and GRO-α in the supernatants of each CNE2-derived cell line were measured by ELISA (n = 3). **(E)** The number of immune cells recruited by CNE2-ZTA and CNE2-CTR (n = 4). **(F)** and **(G)** After treatment with the supernatants of CNE2-ZTA and CNE2-CTR, the difference in the yield of DCs was examined by CD14 and CD1a expression profile, the proportion of DC-d-Ms was examined by CD14 and CD68 (n = 4). **(H)** The proportion of M1 and M2 subtypes were determined by detecting CD86&CD163 (n = 4). **(I)** Neutralizing antibodies were used to block the recruitment of monocytes to CNE2-ZTA. The proportion of CD14^+^ cells was determined by flow cytometry (n = 4). **(J)** Neutralizing antibodies were used to block the ability of CNE2-ZTA to induce TAMs. The proportion of CD163^+^ cells was determined by flow cytometry (n = 4). Data are presented as the mean±SEM; * P < 0.*05*, ** P < 0.*01*, *** P < 0.*001*, **** P < 0.*0001*, *NS*, *not significant*.

We found that GM-CSF, GRO-α, and IL-8 were upregulated in abortive lytic EBV^+^CNE2 and speculated that the EBV abortive lytic cycle regulates monocytes through up-regulating these monocyte-related cytokines. Thus, we sought to identify the viral gene that regulates the expression of these monocyte-related cytokines. The abortive lytic phase of EBV expresses a series of immediate-early and early lytic genes [23]. We selected eight lytic genes commonly express in nasopharyngeal carcinomas for investigation: BZLF1 (ZTA), BRLF1 (RTA), BALF3, BALF4, BALF5, BILF1, LF1, and LF2. Expression vectors for these viral genes were introduced into CNE2 cells and the changes in monocyte-related cytokines expression were analyzed by real-time PCR. Results showed that transient transfection of ZTA increased the expression of GM-CSF, IL-8, and GRO-α up to three to ten-fold (Fig 4A).

To determine if ZTA is indeed responsible for recruiting monocytes and regulating their differentiation, we constructed a CNE2 cell line that stably expresses ZTA. The expression of ZTA was verified by Western blot (Fig 4B). The ZTA-expressing CNE2 cell line was assayed for cytokine expression profile and monocyte chemotaxis. Cytokine antibody arrays demonstrated that the cytokine expression profiles of CNE2-ZTA remarkably resemble that of abortive lytic EBV^+^CNE2, in which GM-CSF, IL-8, and GRO-α were significantly upregulated (Fig 4C). To confirm that ZTA is the primary regulator for cytokine induction and monocyte chemotaxis, an ELISA assay was performed to compare the regulatory effect of EBV abortive lytic phase and ZTA on the production of GM-CSF, IL-8, GRO-α in CNE2 cells. Results confirmed that both ZTA and EBV lytic phase significantly increased the secretion of GM-CSF, IL-8, and GRO-α similarly (Fig 4D).

In terms of promoting the recruitment of monocytes and monocyte differentiation, CNE2-ZTA exhibited a profound chemotactic ability on monocyte differentiation (Fig 4E), significantly reduced the generation of DCs, and up-regulated the proportion of DC-d-Ms. (Fig 4F and 4G). In addition, the CM of CNE2-ZTA distinguishably decreased the ratio of M1 and doubled the proportion of CD163^+^TAMs in Mφs differentiation system (Fig 4H). Similarly, ZTA was also significantly up-regulated in HK-1-EBV^+^ Day7 (S5A Fig). HK-1 cells with ZTA stable expression could induce the expression of monocyte-related cytokines, promote monocyte chemotaxis, and regulate their differentiation towards TAMs and away from DCs (S5B-S5G Fig).

Our result above revealed that ZTA in NPC cells induces the expression of GM-CSF, IL-8, and GRO-α, which is consistent with the previous report that ZTA can promote the secretion of IL-8 [35] and GM-CSF [36]. The regulatory role of ZTA on GRO-α induction was found for the first time. To identify the function of each cytokine in consequent monocyte chemotaxis and targeted differentiation, we utilized neutralizing antibodies against these cytokines in a Transwell recruitment assay with CNE2-ZTA cells. We found that the neutralizing antibodies to IL-8 and GRO-α significantly inhibited chemotaxis of monocytes toward CNE2-ZTA (Fig 4I). In addition, CNE2-ZTA CM was able to induce the differentiation of TAMs, while the addition of the neutralizing antibodies against GRO-α and GM-CSF significantly reduced the expression of CD163^+^TAMs. IL-8 neutralizing antibody had no apparent effect on the differentiation of CD163^+^TAMs (Fig 4J). The effect of neutralizing antibodies on DCs differentiation was not obvious, thus the mechanism of ZTA inhibiting DC differentiation remains to be further explored. Our results demonstrated that ZTA promotes the recruitment of monocytes through IL-8 and GRO-α, while ZTA induces the differentiation of TAMs through GRO-α and GM-CSF.

**Fig 5:**
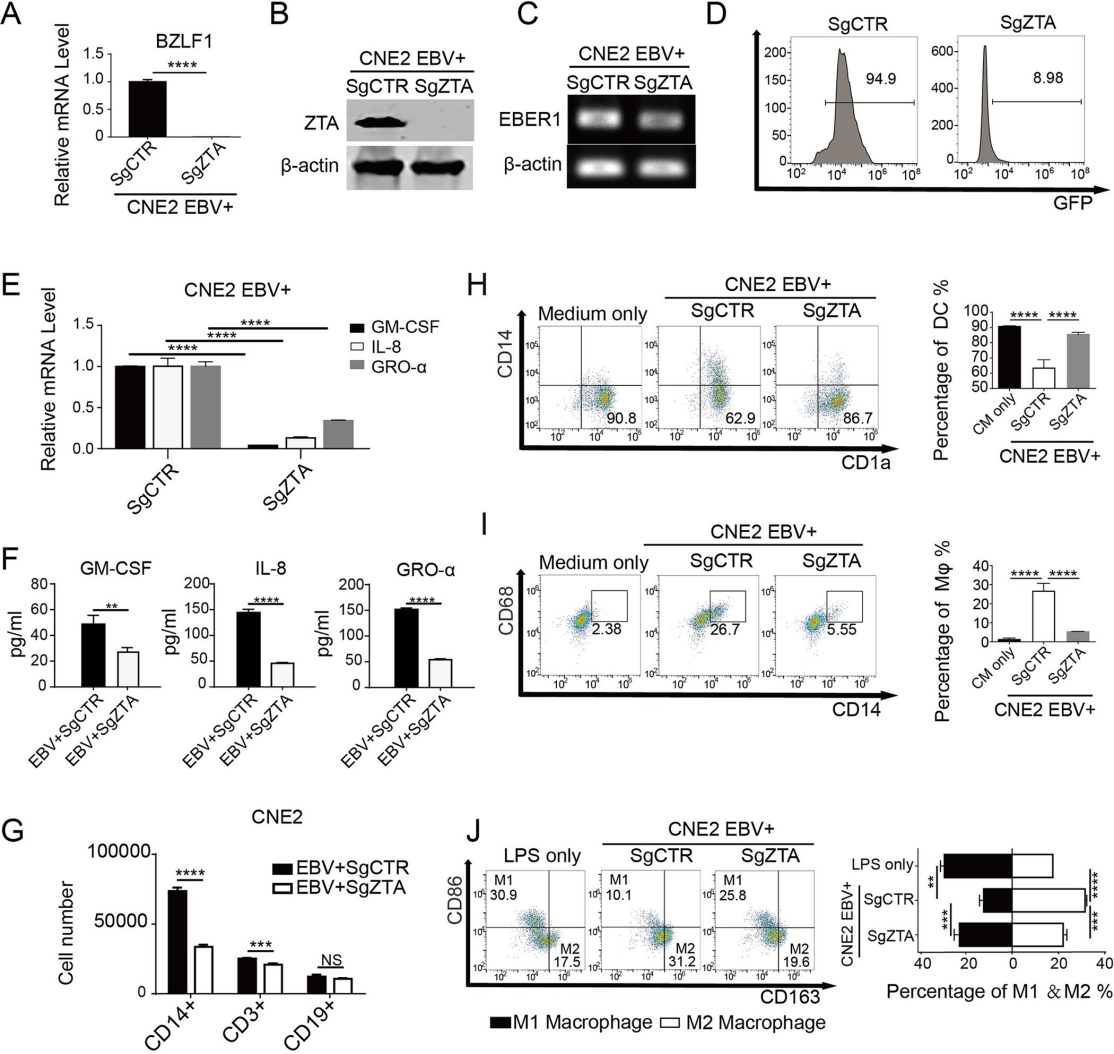
ZTA Knockout EBV lost the capacity of recruiting and polarizing monocyte. **(A)** and **(B)** The CRISPR/Cas9-mediated knockout efficiency of ZTA was estimated by measuring ZTA mRNA expression (n = 3), as well as ZTA protein levels. **(C)** EBER1 expression levels in the knockout and control cells were quantified by RT-PCR. **(D)** The GFP profiles of EBV^+^CNE2 after ZTA deletion were detected by flow cytometry. **(E)** and **(F)** The mRNA expression levels of GM-CSF, IL-8, GRO-α in ZTA knockout and control EBV^+^CNE2 cells were detected by real-time PCR (n = 3), the concentrations of GM-CSF, IL-8 and GRO-α in the supernatants were measured by ELISA (n = 3). **(G)** The number of immune cells recruited by CNE2-EBV^+^SgCTR and CNE2-EBV^+^SgZTA (n = 4). **(H)** and **(I)** After treatment with the supernatants of CNE2-EBV^+^SgCTR and CNE2-EBV^+^SgZTA, the yield of DCs was examined by CD14 and CD1a expression profile. The proportion of DC-d-Ms was examined by CD14 and CD68 (n = 4). **(J)** M1 and M2 proportions were identified by CD86 and CD163 expression (n = 4). Data are presented as the mean±SEM. * P < 0.*05*, ** P < 0.*01*, *** P < 0.*001*, **** P < 0.*0001*, *NS*, *not significant*.

We also employed a loss-of-function approach to confirm the role of ZTA in regulating monocytes. CRISPR/Cas9-mediated knockout was used to establish ZTA-deficient EBV^+^CNE2 (CNE2-EBV^+^SgZTA) [37]. The efficiency of ZTA knockout was determined by real-time PCR and Western (Fig 5A and 5B). The ratio of GFP-positive CNE2 fell from 94.9% to 8.98% in the absence of ZTA, while the expression of EBER1 in the CNE2-EBV^+^SgZTA group was only slightly decreased, proving that most of EBV entered the latency rather than being in lytic phase in the absence of ZTA (Fig 5C and 5D). Then, we examined whether the ability of CNE2 to regulate monocyte chemotaxis and differentiation was compromised after ZTA is knocked out. We found that (i) the expression of GM-CSF, IL-8, and GRO-α significantly decreased in CNE2-EBV^+^SgZTA, compared with CNE2-EBV^+^SgCTR (Fig 5E and 5F); (ii) the proportion of CD14^+^ monocytes recruited by CNE2-EBV^+^SgZTA was decreased, revealed in a Transwell recruitment experiment (Fig 5G); (iii) the CM from CNE2-EBV^+^SgZTA resulted in a marked increase in the proportion of DCs and a decrease in the ratio of DC-d-Ms, compared to the CM from CNE2-EBV^+^SgCTR (Fig 5H and 5I). Furthermore, the capability of CNE2-EBV^+^SgCTR to decrease the M1 ratio and increase the M2 proportion was lost in CNE2-EBV^+^SgZTA, and the proportions of M1 and M2 returned to normal levels (Fig 5J). Similar results were obtained in the EBV^+^HK-1 cells where ZTA was knocked out (S6A-S6J Fig). In conclusion, ZTA is the viral mechanism by which EBV abortive lytic cycle regulates monocytes.

### EBV abortive lytic cycle promoted NPC angiogenesis and invasion via inducing TAMs differentiation

**Fig 6:**
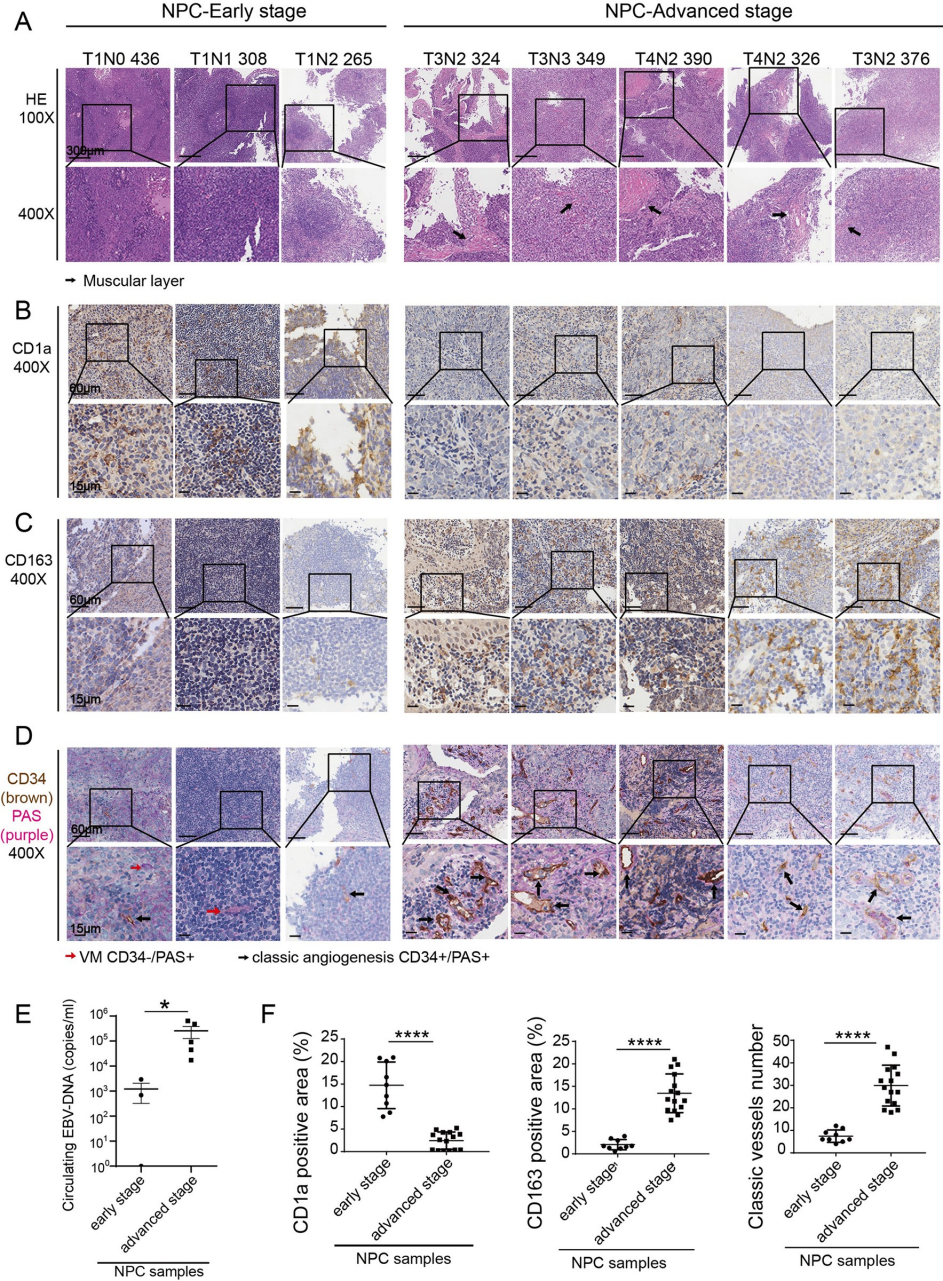
EBV activation, TAMs infiltration and angiogenesis in advanced NPC samples. **(A)** Clinical NPC samples were analyzed by hematoxylin and eosin (H&E) staining (100X 400X). **(B) and (C)** Representative images of tumor showing CD1a^+^ DCs and CD163^+^ TAMs. Cells were immunostained with the corresponding antibodies (400X). **(D)** Representative images of tumor showing PAS (Purple) expression and CD34^+^ cells (Brown) (400X). Black arrows indicate classical angiogenesis (PAS^+^/CD34^+^). **(E)** EBV-DNA titer in plasma from circulating blood, obtained from the hospital information system. **(F)** The positive area of each stain was quantified using ImageJ in randomly chosen 400X fields (n = 3 per sample).Data are presented as the mean±SEM; * P < 0.*05*, ** P < 0.*01*, *** P < 0.*001*, **** P < 0.*0001*, *NS*, *not significant*.

To investigate the clinical significance of the EBV lytic cycle in advanced NPC, clinical samples, including three in the early TNM stage and five in the advanced stage, were obtained and examined with immunohistochemical analysis. H&E stains exhibited that in advanced NPCs, tumor cells invaded deeply into the submucosal muscular layer, and the tumor tissue structure is relatively loose, with many lacunar structures (Fig 6A). On the other hand, lower CD1a^+^ DC cell infiltration and CD163^+^ TAMs infiltration were observed in advanced NPC compared with early-staged NPC (Fig 6B and 6C), which validated our conclusion that lytic or abortive lytic EBV exhibited superior capacity in promoting monocyte differentiation toward TAM-like phenotype and away from DCs.

The observed increased lacunar structures in advanced NPC might represent abnormal angiogenesis. This notion was confirmed by double staining of CD34 and PAS (CD34^+^/PAS^+^), which identified classic angiogenesis [38]. CD34 marks angiogenic tip endothelial-like cells, and PAS (Periodic Acid-Schiff stain) emphasizes the capillary basement membrane. We also observed that cells lack CD34 markers in some areas, but PAS are positive, indicating vascular mimicry (VM). Therefore, a large amount of neoangiogenesis was seen in the advanced NPC samples, which was not seen much in the early-staged NPC samples (Fig 6D). There were articles in which EBV-DNA load was used as an index to detect EBV lytic reactivation [39,40]. On the other hand, the highest titers of anti-ZEBRA(ZTA) IgGs were associated with high viral loads [41]. Therefore, we selected EBV-DNA load as an indicator of the degree of EBV reactivation in NPC patients. The EBV titer in the plasma of circulating blood confirmed the presence of significant EBV lytic activation in advanced NPC samples (Fig 6E), The positive areas of CD1a and CD163 and the number of blood vessels in each sample were quantified by Image J (Fig 6F). Furthermore, we used Spearman Rank Correlation Test to explore the correlation between EBV-DNA load and TAM/vessel density. Results proved that higher EBV titers were accompanied by stronger TAM infiltration and blood vessel density (S7 Fig).

**Fig 7:**
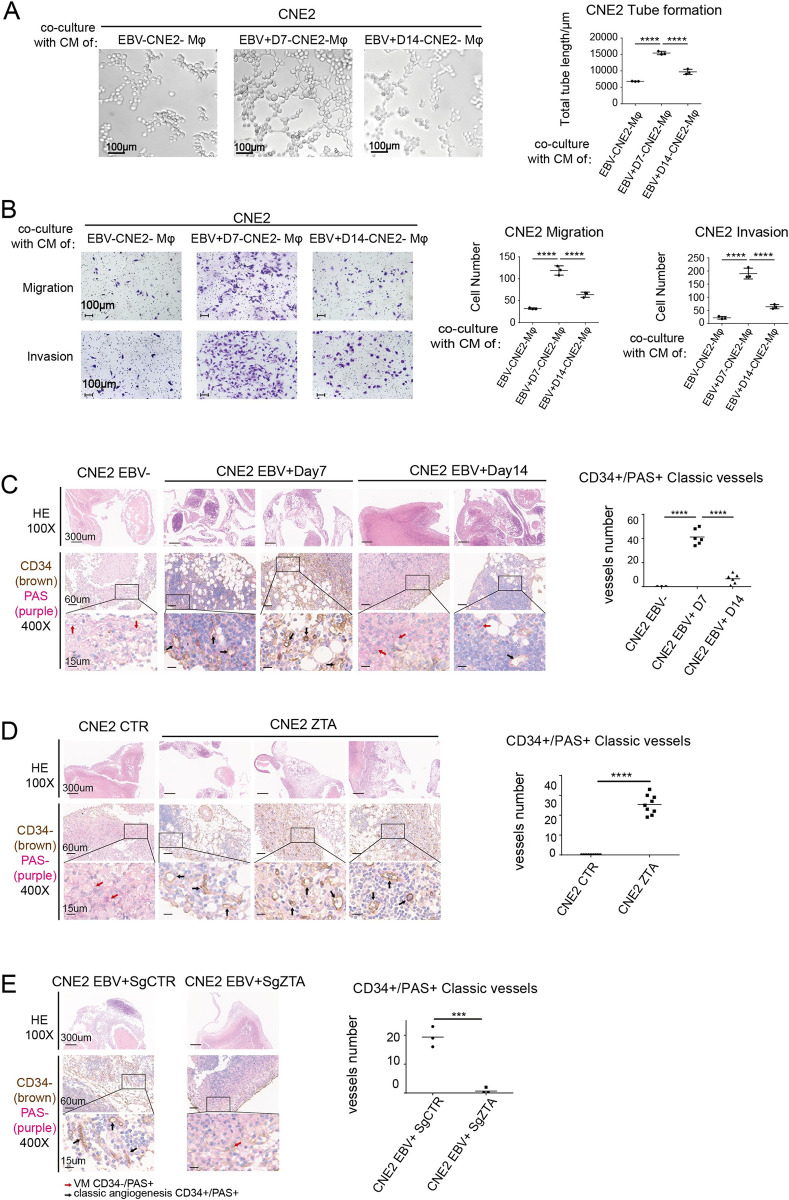
TAMs induced by EBV abortive lytic cycle promote NPC angiogenesis and invasion. **(A)** Effect of macrophage supernatants from different groups on angiogenesis of CNE2 analyzed by Matrigel tube assay *in vitro* (n = 3). **(B)** Effect of macrophage supernatants from different groups on migration and invasion abilities of CNE2 cells assayed by Transwell migration assay and Transwell-Matrigel invasion assay (n = 3 per sample). **(C) to (E**) Tumors xenografts in the abdominal cavity of mice were analyzed by hematoxylin and eosin (H&E) staining (100X) and vascular immunohistochemical staining. Representative images of tumor showing PAS (Purple) expression and CD34^+^cells (Brown) (400X) were presented. Red arrows indicate VM channels (PAS^+^/CD34-), black arrows indicate classical angiogenesis (PAS^+^/CD34^+^). Quantification of classical angiogenesis determined by microscopy with 400X magnification in randomly chosen fields (n = 3 per sample). Data are presented as the mean±SEM; * P < 0.*05*, ** P < 0.*01*, *** P < 0.*001*, **** P < 0.*0001*, *NS*, *not significant*.

Studies have shown that the infiltration density of TAMs in tumor tissue is correlated to tumor cell proliferation, blood vessel density and poor clinical prognosis [42]. That prompted us to ask whether infiltration of TAMs due to EBV abortive lytic cycle contributes to angiogenesis and invasion of NPC. We hypothesized that the strong invasiveness and angiogenesis shown in the advanced NPC samples were related to the infiltration of TAMs induced by the EBV lytic cycle. To validate this hypothesis, we used CM of CNE2-EBV^-^, CNE2-EBV^+^DAY7, and CNE2-EBV^+^DAY14 to induce M2 macrophage differentiation, respectively. As expected, CNE2-EBV^+^DAY7 had the most robust ability to induce TAMs differentiation. Then, the supernatant of these macrophages (Mφ-CM) was collected and used it to culture EBV-negative CNE2. After a week, we examined the capacities of these CNE2 in tube formation, migration, and invasion. The results showed that the CNE2 cultured in the CM of CNE2-EBV^+^DAY7-induced-Mφ exhibited significantly enhanced tube-forming capacity in comparison to the CM of CNE2-EBV^-^ and CNE2-EBV^+^DAY14 induced-Mφ (Fig 7A). Similarly, Transwell migration and invasion assays also showed that TAMs induced by the EBV lytic cycle had the most tumorigenic ability, promoting migration and invasion of NPC cells (Fig 7B).

To further verify the ability of the EBV lytic cycle to promote NPC angiogenesis and invasiveness *in vivo*, EBV-negative CNE2 and CNE2 with latent EBV or abortive lytic EBV were inoculated into C57 BL/6 intraperitoneally. After seven days, the tumor xenografts were detached from intra-abdominal adipose tissues and analyzed by immunobiological analysis. H&E staining showed that the tumor xenografts in the CNE2-EBV^+^DAY7 group presented abundant reticular structures, suggesting invasion and colonization of tumor cells in adipose tissue in CNE2-EBV^+^DAY7 tumor xenografts. The tumor xenograft structure of CNE2-EBV^-^group was compact without reticular structure; most cells were dead, and the nuclear structure was vague and loose with only a few living cells seen on the outer edge. The tumor xenografts of the CNE2-EBV^+^DAY14 group exhibited a small number of reticular structures (Fig 7C). H&E staining confirmed that tumors formed by abortive lytic EBV^+^CNE2 exhibited most robust invasive properties. ZTA and EBNA1 were analyzed by immuno-histochemical staining to confirm the EBV life cycle in these xenografts, showing the highly ZTA expression in the CNE2-EBV^+^DAY7 group, while EBNA1 was detected in every EBV-positive xenograft (S8 Fig).

Angiogenesis in tumor xenografts was examined by double staining the samples for CD34 and PAS. The results indicated that the tumor xenografts in CNE2-EBV^+^DAY7 group had a large amount of classical angiogenesis, manifested by lots of lacuna (CD34^+^/PAS^+^) in these xenografts, and red blood cells were observed in some cavities, while there was only a small amount of vascular mimicry in the tumor xenografts of CNE2-EBV^-^ and CNE2-EBV^+^DAY14 groups (Fig 7C). CNE2 with ZTA overexpression also exhibited the most robust angiogenesis and invasive abilities likewise (Fig 7D). Angiogenic capabilities of CNE2-EBV^+^ was attenuated in CNE-EBV^+^DAY7 where ZTA had been knocked out (Fig 7E). Histochemical staining of mice xenografts supports the conclusion that NPC with abortive lytic EBV exhibited most robust invasion and angiogenesis abilities.

Taken together, our result confirmed that CNE2 in EBV abortive lytic cycle could induce TAMs differentiation, that in turn significantly enhances CNE2 invasion and angiogenesis, thus promoting tumor progression.

## Discussion

The life cycle of EBV include latency and lytic phases. Type II latency is the most common EBV transcriptional pattern in NPC [43]. However, as carcinoma progresses, lytic genes begin to express. The role of the EBV lytic cycle in advanced NPC has been elusive. In this study, we demonstrated that EBV abortive lytic cycle promotes angiogenesis and invasion of NPC by regulating the immune microenvironment. First, analysis of clinical NPC samples confirmed the presence of EBV lytic activation and immunosuppression in advanced NPC, as well as abnormal angiogenesis and invasiveness. The NPC cells with EBV abortive lytic cycle are found to recruit monocytes and induce their differentiation towards TAMs and away from DCs. Second, EBV immediate-early protein ZTA is the key viral mechanism of EBV lytic cycle regulating monocyte chemotaxis and differentiation toward TAM. Third, TAMs induced by EBV abortive lytic cycle, in turn, promote tumor cell angiogenesis and invasion. Our study revealed the involvement of EBV abortive lytic cycle in TAM formation, leading to abnormal angiogenesis and invasiveness in advanced NPC.

In contrary to traditional wisdom that virally associated tumors are mainly attributed to the latent cycle of a tumor virus but not to the lytic phase simply because a lytic virus in general leads to lysis and death of host cells, increasing evidence suggests that EBV lytic cycle plays roles in EBV-mediated tumorigenesis. The evidence that supports this notion is as follows. (i) Transcriptomic studies of EBV-associated NPC and lymphomas always revealed coexpression of EBV lytic genes with cellular cancer-associated pathways [6]. (ii) High EBV titers in the circulating blood of NPC patients are positively correlated with NPC recurrence, metastasis, and mortality [44]. High titers of anti-ZTA IgG in the serum of NPC patients are associated with poor clinical outcomes [45]; (iii) Despite similar infection levels, the ability to lytic replication-incompetent EBV particles to cause lymphomagenesis is impaired compared with wild-type virus [46]. In addition, it was also found that aciclovir treatment, which prevented viral genome replication but not lytic gene expression, did not prevent EBV-associated lymphomagenesis. This observation suggested that the lytic gene expression, not viral genome replication or virion production, may play a significant role in tumorigenesis [46]. Although a complete cycle of EBV lytic replication in a small percentage of infected cells may contribute to tumor development by producing and disseminating viral particles to more host cells, the restricted expression of lytic genes without producing infectious particle, designated as “abortive lytic cycle”, has emerged as a predominant mechanism for viral lytic cycle-associated cancer development [47,48]. Our current study provided further evidence for this theory and uncovered novel insights into how EBV lytic cycle facilitates NPC late-stage development.

In EBV lytic replication, ZTA functions as a switch protein because it initiates EBV lytic gene expression cascade to switch EBV from latency into lytic life cycle. As a transcriptional activator, ZTA also activates many host cell genes. ZTA is considered to have significant immunosuppressive functions. ZTA is required for tumor formation in SCID mice [46]. ZTA directly inhibits the interferon response signaling pathway [49] and MHC II presentation [50]. It is reported that ZTA induces the secretion of IL-8 by host cells and thus promoting the recruitment of granulocytes by NPC [35]. ZTA can also promote the secretion of IL-10 [51], which plays an essential role in tumor immune evasion and tumor-promoting immunity. ZTA also induces GM-CSF and Cox-2 expression and thus promotes monocytes secreting IL-10 [36]. As a crucial viral mechanism to regulate monocytes, we investigated the role of ZTA in regulating monocyte differentiation toward TAMs. Knockout of ZTA significantly inhibited the recruitment of monocytes and the differentiation of TAMs, and attenuated tumor invasion and angiogenesis *in vitro* and *in vivo*. In addition to IL-8 and GM-CSF, we also found that GRO-α was also regulated by ZTA, which may lead us to explore more functions of ZTA.

CCL5, ranked fourth in Fig 2F, was not included in our study for the following reasons: Firstly, while EBV infection does promote the expression of CCL5, the expression difference between the 7^th^ day and the 14^th^ day of CNE2 infection is slight, although the difference is statistically significant (S9A Fig). Second, when we analyzed the host gene expression differences between advanced and early clinical samples, we found that CCL5 expression was down-regulated in advanced clinical samples (EBV was more active in this group of samples), as seen in S9B Fig. Besides, some studies proposed that CCL5/RANTES is regulated by EBV latent membrane protein LMP1 [52,53]. The above content suggests that CCL5 expression was not associated with EBV activation in advanced NPC.

One of the big challenges in studying abortive lytic replication is the lack of cell lines that undergo an incomplete lytic cycle without viral particle being produced and released. *In vitro*, EBV is always latent in B cell lines, expressing only a few latent genes. The lytic cycle can be artificially induced by chemical stimuli such as TPA (12-O-Tetradecanoyl-phorbol-13-acetate) and NAB (sodium butyrate), which can drive EBV into complete lytic cycle in B cells as well as NPC cells [54]. However, the lytic cycle-inducers also change the inflammatory signaling system of NPC cells, masking the immunosuppressive function of the lytic cycle [55]. On the other hand, cell death and disintegration caused by the release of EBV virions also induce a robust inflammatory response. In our research, both EBV lytic inducers and complete EBV lytic cycle are inappropriate for exploring the relationship between EBV lytic cycle and tumorigenesis. It is known that EBV will enter a brief lytic cycle after infecting B cells, which only expresses early lytic genes to promote DNA replication but does not produce viral particles. This eight-day window period is called the pre-latent abortive lytic cycle, and then EBV enters total latency [56]. We found the same phenomenon in epithelial cells: in EBV-infected NPC epithelial cell lines on day 7, intracellular EBV maintained abortive lytic activation. Compared with TPA-treated cells, NPC cell lines in EBV pre-latent lytic cycle have optimal growth status, low apoptosis rate, and low immunogenicity, accurately mimicking the activation state of EBV abortive lytic cycle in advanced NPC. This system, including NPC cells on Day 7 (pre-latent abortive lytic phase) and NPC cells on Day14 (true latent phase), was successfully used in our study, which provided novel insights into the role of EBV abortive lytic replication in NPC pathogenesis *in vitro* and *in vivo*.

**Fig 8:**
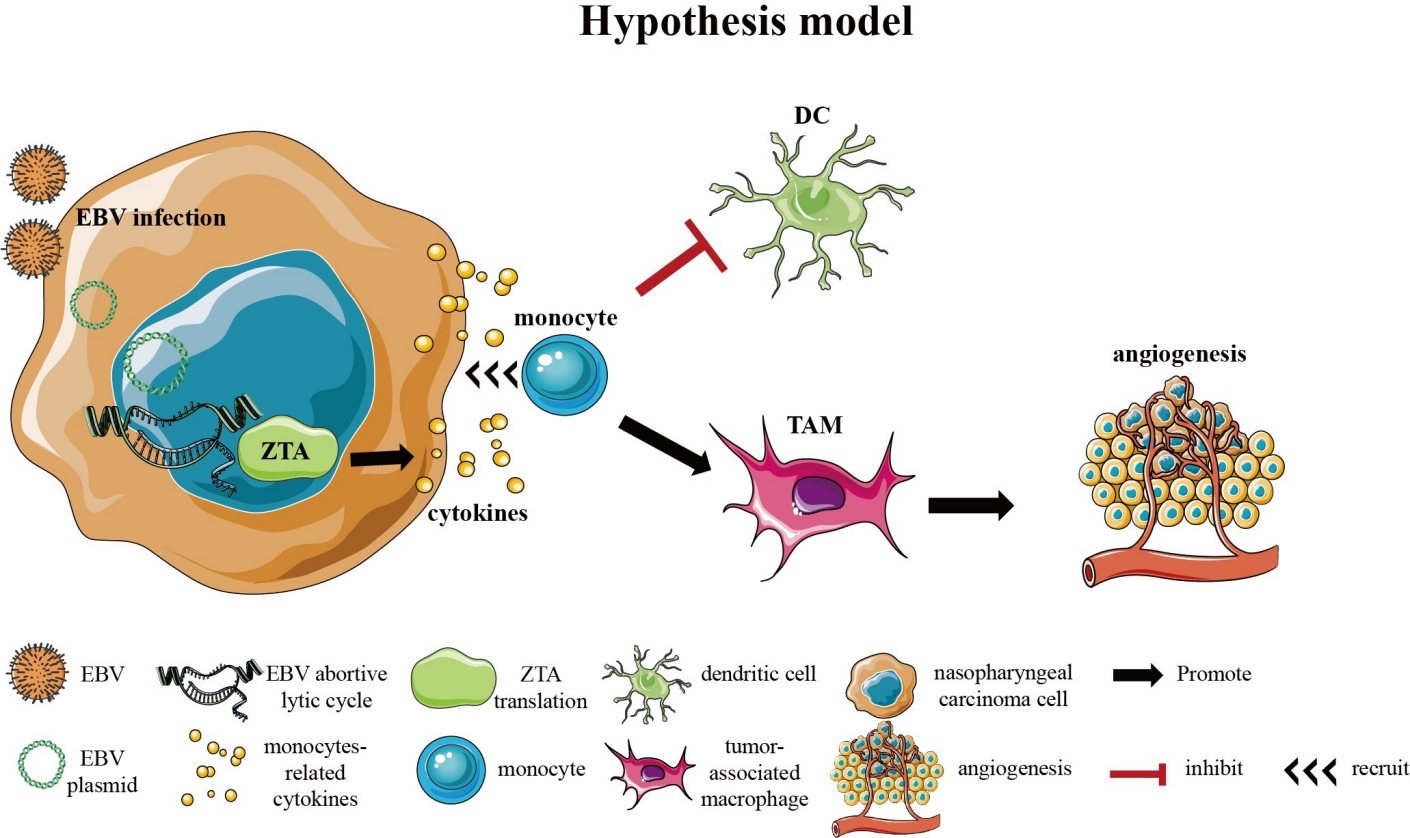
Schematic model for the role of EBV-abortive lytic cycle in modifying monocytes to establish the tumor-promoting microenvironment that promotes NPC late-stage progression.

Prior to our study, reports have shown that EBV lytic cycle or ZTA facilitates the secretion of proangiogenic factor VEGF and cytokines IL-6, -8, -10, and -13 to promote angiogenesis in NPC. Through VEGF, EBV-infected cells recruit monocytes and activate monocytes into TAMs, ultimately promoting tumor metastasis [14]. In the NPC tumor microenvironment, TAM infiltration was found closely related to the poor prognosis of NPC. However, the relation between the infiltration of TAMs and the EBV lytic cycle in advanced NPC was unknown. Our study elucidated the contribution of EBV abortive lytic cycle in the progression of advanced NPC by regulating the differentiation of TAMs and promoting angiogenesis in advanced NPC. A model for lytic EBV regulating monocyte differentiation and functional consequence in late-stage NPC is illustrated in Fig 8.

Tumor metastasis and recurrence, which is responsible for the vast majority of cancer deaths, depends on angiogenesis or lymphangiogenesis in tumor tissues. Therefore, angiogenesis plays an essential role in tumor progression. The tumor cells invade the lumen, spread, and settle with the circulation of body fluid, thus producing new metastatic or recurrent lesions [57]. Local hypoxia and an acidic environment in advanced NPC will lead to the activation of EBV lytic cycle, which will promote the infiltration of TAMs and thus result in the angiogenesis and invasion of the tumor. The finding that EBV abortive lytic cycle induce TAMs differentiation, that in turn significantly enhances NPC invasion, angiogenesis, and tumor progression, offers a new strategy to treat advanced NPC through intervening EBV activation in advanced NPC to reduce the metastasis and recurrence rate. In addition, intensity-modulated therapy has been the primary method in treating nasopharyngeal carcinoma [58], still radiotherapy and chemotherapy not only kill tumor cells, but also activate the EBV lytic cycle and thus leading to metastasis and recurrence of tumors. On the other side, some reports suggested that ZEBRA (ZTA) can be released into the bloodstream by infected cells and can potentially penetrate target cell through its cell-penetrating domain [59,60]. In conclusion, we think that an antibody cocktail targeting ZTA or GM-CSF, IL-8, and GRO-α may block the angiogenesis in NPC, thus reducing metastasis and recurrence and improving the quality of life in patients with advanced NPC or patients after radiotherapy, but further clinical research is warranted.

## Materials and methods

### Ethics statement

The use of NPC clinical samples in the research were approved by the Sun Yat-sen University Cancer Center (Approval No. YB2016-076), in accordance with International Conference on Harmonization Good Clinical Practice (ICH GCP) guidelines, government regulations, and laws. Written informed consent was provided by study participants. All the Patient’s clinical characteristics and EBV titer in the plasma were obtained from the hospital information system (HIS) of Sun Yat-sen University Cancer Center. The collection and the use of Peripheral Blood Mononuclear Cells (PBMCs) in our research were approved by the Medical Ethics Review Board of Sun Yat-sen University (approval no.2018ZX10302-103). Written informed consent was provided by study participants. The animal experiments in this study were approved by the Animal Ethics Review Board of Sun Yat-sen University (approval no. SYSU-IACUC-2021-000478) and carried out strictly following the Guidance suggestion of caring laboratory animals, published by the Ministry of Science and Technology of the People’s Republic of China.

### Cells and culture conditions

CNE2, HK-1, and Akata cell lines were provided by Dr. Mu-Sheng Zeng at Sun Yat-sen University Cancer Center. CNE2 as well as its derived cell lines were grown in RPMI 1640 medium containing 5% fetal bovine serum and 1% penicillin/ streptomycin. HK-1 and its derived cell lines, and Akata cell line with recombinant EBV were maintained in RPMI 1640 containing 10% FBS. Cell lines containing rEBV were cultured in the presence of G418 (700 g/mL, GIBCO) every 2–3 generations to maintain EBV episomal genome. Human embryonic kidney (HEK) 293T cells (ATCC) were cultured in Dulbecco’s Modified Eagle’s Medium (DMEM) supplemented with 10% FBS and 1% penicillin/ streptomycin. All cells were cultured in a humidified 5% CO_2_ atmosphere at 37°C.

### Establishment of EBV-positive NPC cell lines

GFP fluorescein gene carried by recombinant EBV is located on the BXLF1 open reading frame. After activation of EBV lytic cycle, GFP began to express in host cells. rEBV-infected Akata was suspended in 1640 medium containing 3% FBS to a density of 5×10^6^/mL. IgG (goat anti-human, 8‰) was added to the medium to induce EBV lytic replication and virion production. CNE2 was planted on T25 flask and cultured overnight. Then the supernatant of CNE2 was replaced by a mixture of 2 ml activated rEBV-Akata cell suspension and additional 3ml completed RPMI 1640 medium. Half of the supernatant was changed every other day. After co-cultured for 2–4 days, Akata was washed away with PBS, GFP-positive EBV^+^ CNE2 was obtained after G418 selection.

### Plasmids and cell transfection

Plasmid pEGFP-C3(#VT1109) was purchased from Youbio, China. A series of EBV lytic genes were cloned into the EcoRI and XbaI site of pEGFP-C3 vectors using One Step Cloning Kit (#C112-02, Vazyme) respectively. The PCR fragments of EBV lytic genes were amplified from total DNAs prepared from EBV positive NPC cell line C666-1 and the primers listed in S1 Table.

For cell transfection: After trypsin digestion, CNE2 was suspended and planted on a 6-well plate and cultured overnight at 37°C until the cell density reached 70%. peGFP-C3 was transfect into CNE2 (2 μg/ well). 48 hours later, cells with GFP expression were observed with Zeiss observer Z1 and the virogene expression was detected by real-time PCR.

### Establishment of NPC cell lines stably expressing ZTA

Plasmid pLVX-EF1α -LUC-N1 (#VT9009) was purchased from Ubio China. PCR fragment of BZLF1 (ZTA) was amplified from the total DNA of C666-1 cells with following primers: BZLF1-F-BAMHI: 5’-GGTACCGCGGGCCCGGGATCCCCACCTTTGCTATCTTTGCTGA-3’, BZLF1-R-XBAI: 5’-TACCCGGTAGAATTATCTAGAAACAAGTGCGATGGCGGTA-3’. BZLF1 fragment was subcloned into the BAMHI and XbaI sites of plVX-EF1 α-Luc-N1 vector using the One-step Cloning Kit (#C112-02, Vazyme). The pLVX-EF1α-ZTA-N1 vector and control lentiviral vector were applied to package lentiviral particles. Lentiviral vectors were co-transfected with packaging plasmids psPAX2 (#12260, Addgene) and pMD2G (#12259, Addgene) into HEK293T cells in a ratio of 4:3:1, followed by incubation for 72 hours. The medium containing lentiviruses was harvested and subjected to ultracentrifuge for lentiviruses concentrations. Lentiviruses were used in infected NPC cell lines and followed by puromycin selection for 1 week. CNE2-ZTA and CNE2-CTR cell lines were obtained.

### CRISPR-Cas9-mediated knockout of EBV BZLF1 expression

Guide sequences (gRNAs) were designed to target the 5’ and 3’ regions of BZLF1 using an online CRISPR design tool (http://crispr.mit.edu). The sequences for 5’ and 3’ regions are as follows:

BZLF1-SG1-F:5’-CACCGCAACTGACTAACCAAGCCGG-3’; BZLF1-SG1-R:5’-AAACCCGGCTTGGTTAGTCAGTTGC-3’; BZLF1-SG2-F:5’-CACCGGATTTGGCAGAAGCCACCTG-3’; BZLF1-SG2-R:5’-AAACCAGGTGGCTTCTGCCAAATCC-3’;

The gRNA sequences were subcloned into the BsmBI restriction site of CRISPR/Cas9 vectors lentiCRISPR v2 (#52961, Addgene). Lentivirus was produced by triple transfection of 293T cells with the sgRNA expression LentiCRISPR-v2 vector and the packaging plasmids psPAX2 and pMD2G at a ratio of 4:3:1. These two gRNA/ Cas9-expressing lentivirus, were transduced to EBV positive CNE2, followed by puromycin selection for 1 week, EBV^+^CNE2-sgZTA and EBV^+^CNE2-sgCTR cell lines were obtained.

### Real-time PCR

Total RNA was extracted using Ultrapure RNA Kit (#CW0581, CWBIO) and converted into cDNA using the reverse transcription kit (Promega) according to the manufacturer’s instructions. The relative mRNA expression levels were quantified by real-time PCR using LightCycler 480 SYBR Green I Master (Roche) with specific primers for the genes of interest and normalized to GAPDH. The primer sequences used for real-time PCR were listed in S2 Table. All real-time PCR was done in triplicate.

### EBER1 Reverse-transcriptional PCR (RT-PCR)

The RNA of EBV-positive cell lines was extracted, and reverse transcribed into DNA fragments. Two pairs of primer were used to amplified DNA sequence of EBER1 and β-actin. Prepare an agarose gel by combining the agarose and water. Add at least one-tenth volume of 10X agarose gel loading dye (#9157, Takara) to each DNA sample, mix, and load into the gel wells. Electrophorese the gel at 150–200 mA until the required separation has been achieved. Visualize the DNA fragments on a long wave UV light box and photograph with a Polaroid camera.

the sequence of Primer is as follows:

EBER1-F:5’-CCCAGATCTAGGACCTACGCTGCCC-3’;

EBER1-R:5’-CCCAAGCTTAAAACATGCGGACCACCAGC-3’;

β-actin-F:5’-TGTTACCAACTGGGACGACA-3’;

β-actin-R 5’-CTGGGTCATCTTTTCACGGT-3’.

### Western blot

Preparation of whole-cell protein lysates and western blot analysis were performed as described previously [5]. The whole cell extract of 40 μg protein was resolved in SDS-PAGE and transferred to a nitrocellulose membrane. The membranes were blocked with 5% non-fat milk/PBS for 30 minutes and incubated with primary antibodies overnight at 4°C. Anti-β-actin (#A5441, Sigma) and anti-EBV ZEBRA (BZLF1) (#sc-53904, Santa Cruz) were used as the primary antibodies in this study. Anti-IR Dye 800 or Dye 680 anti-rabbit or anti-mouse IgG antibodies (LI-COR Biosciences) were used as the secondary antibodies. An Odyssey system (LI-COR Biosciences) was used for detecting the proteins of interest.

### Human-monocyte isolation and differentiation

CD14^+^ monocytes were isolated from healthy human peripheral blood mononuclear cell (PBMC) by EasySep Human CD14 Positive Selection Kit II (#17858, Stemcell) and cultured in RPMI 1640 containing 10% FBS.

Culture of dendritic cells: isolated monocytes were suspended to a density of 1×10^6^/mL, 400 μl/ well planted in 48 well, GM-CSF (50 ng/ mL, Peprotech) and IL-4 (20 ng/ mL, R&D) were added into the system, and half of the medium was changed every other day. After 4–5 days, dendritic cells were obtained. On the fourth day, 50% of the medium was replaced with NPC cell supernatant of different groups. DC yield was determined using CytoFLEX flow cytometer after 48 hours.

Culture of macrophages: monocytes were cultured with M-CSF (10 ng/mL, Peprotech) for 4–5 days to obtain macrophages. On the fourth day, the supernatant was thoroughly discarded to completely remove M-CSF. Macrophages were cultured in a mixture of 1640 completed medium and NPC cell supernatants of different groups in 1:1 ratio, LPS (1 μg/ mL) was added into the system as macrophage activator. The proportion of M1/M2 macrophage was determined by CytoFLEX after 48 hours.

### Flow cytometry

Cells (1x10^6^) were suspended in 100 μl PBS and incubated with flow antibody (5μl/ 100 μl) in the dark for 20 min. Cells were resuspended in PBS and analyzed using CytoFLEX flow cytometer.

CD68-staining: cells were re-suspended in PBS and IC fixation in 1:1 ratio and incubated at room temperature for 2 hours. IC fixation (#00-8222-49, eBioscience) was washed away and cells were re-suspended in membrane-breaking solution (#00-8333-56, eBioscience) with CD68 antibody (10 μg/mL), and incubated cells in dark at room temperature for 90 min. CD68 expression was detected using CytoFLEX flow cytometer.

The flow antibodies used in this study are as follows. APC anti-human CD14 (#17-0149-42, eBioscience); APC Anti-human-CD3 (#17-0037-42, eBioscience); APC-eFluor780 Anti-human-CD19 (#47-0199-41, eBioscience); PerCP/Cyanine5.5 anti-human CD1A (#300129, Biolegend); PE anti-human CD68 (#12-0689-41, eBioscience); PE anti-human CD86 (#12-0869-41, eBioscience); APC anti-human CD163 (#333609, Biolegend).

### Transwell PBMC recruitment assay

CNE2-derived cells were digested into suspension (4×10^5^/mL). 1 mL of cells were transferred to the bottom chamber of Transwell and incubated at 37°C with 5% CO_2_ overnight. 1 mL PBMC suspensions (5×10^6^ cells) was added to the upper chamber (6 well, 3μm) of the transwell insert (#3422, Corning). Then cells were incubated for 18–24 hours. The supernatant of the bottom chamber was collected and the suspended cells were subjected to flow cytometry for detecting the proportion of immune cell types.

### Animal study

Male C57 BL/6 mice (6–8 weeks old) were purchased from the Laboratory Animal Center of Sun Yat-Sen University and the animals were handled in accordance with the institutional guidelines. CNE2 was injected into the abdominal cavity of the mice (1x10^7^ cells per injection). After 7 days, the abdominal cavity of mice was exposed, and tumor xenografts were removed for immunohistochemical analysis.

### Cytokine antibody array and ELISA

Cytokine antibody array: cytokine profiles of CNE2-pLVX-CTR and CNE2-pLVX-ZTA were determined by analyzing their culture media using the Human Cytokine Array C5 Kit (#AAH-CYT-5-2, RayBiotech) according to the manufacturer’s instruction. The results of the cytokine array were detected by Chemiluminescence detector (ChemiDoc XRS ^+^), and Image J software was used for quantitative analysis.

ELISA: Secreted cytokines in CNE2 supernatant were quantified by using Human GRO-α ELISA Kit (#E-EL-H0045C, Elabioscience), Human GM-CSF ELISA Kit (#RK00045, abclonal), Human IL-8 ELISA Kit (#EHC008.96, Neobioscience), based on the forward sandwich binding technique, each ELISA experiment was done following the protocol provided by the manufacturer.

### Histological analyses

Clinical nasopharyngeal carcinoma samples and C57 BL/6 tumor xenografts were analyzed by the histochemical stain. Briefly, the paraffin-embedded sections of tumor tissues were deparaffinized and rehydrated. After antigen retrieval with 0.1M citrate buffer (pH 6.0), sections were blocked with 5% BSA for 30 minutes. The tumor samples were incubated with primary antibodies at 4°C overnight. Anti-EBV ZEBRA (BZLF1) (#sc-53904, Santa Cruz), anti-EBV EBNA1 (ab20870, Abcam), anti-CD34(#ZA-0550, ZSGB-bio), anti-CD163(#GB113152, Servicebio), anti-CD1a (#MA5-12526, Invitrogen) were used as the primary antibodies. PAS staining was performed using a PAS Staining Kit (#G1008, Servicebio). Images were acquired using Zeiss observer Z1, the numbers of classic angiogenesis were counted from three randomly chosen fields. Quantification of the positive area of CD1a versus CD163 was performed by the IHC Profiler function of IMAGE J.

### Migration and invasion assays

CNE2 was cultured in the medium containing 30% macrophage supernatant from different groups for a week. Then cell was digested into suspension(5×10^5^/mL). For migration assay, 100 ul of the cell suspensions was added to the upper chamber (24well, 8μm) of Transwell insert (#3422, Corning), and 20% FBS RPMI 1640 medium was added to the bottom chamber. Cells were incubated at 37°C incubator for 18–24 hours. For invasion assay, the upper chamber (24 well, 8 μm) of transwell insert (#3422, Corning) was coated with 50 μl of diluted (1:5) Matrigel. 200 μl of cell suspension was added into the upper layer of Matrigel per well and incubated at 37°C for 18–24 hours. Cells that have invaded to the lower surface of the porous membrane were fixed using Ethanol for 15 minutes and stained by Crystal Violet. The invaded cells were visualization by Zeiss observer Z1.

### In vitro tube formation assay

Forty-eight-well plates were coated with Matrigel (1:1 dilute with 1640 without FBS, 100 μl/well) and incubated at 37°C for 1 hour to promote gel coagulation and avoid bubbles. CNE2 was cultured in the medium containing 30% macrophage supernatant from different groups for a week, and then CNE2 were digested into suspension(3×10^5^/mL). 200 μl cell suspension was added into the upper layer of Matrigel per well. Cells were incubated at 37°C for 2–4 hours and images of tube formation were captured using a ZEISS fluorescence microscope. Image J was used to measure the total length of the tube.

### Cluster analysis of viral genes and host genes

RNA-Seq data of nasopharyngeal carcinoma samples were donated by Dr. Mu-Sheng Zeng at Sun Yat-sen University Cancer Center. Raw reads from RNA-Seq data were uniformly processed and converted to RPKM (reads Per Kilobase of exon model Per Million mapped reads) values [23]. The RPKM of host genes and EBV genes were deposited in the S3 and S4 Tables separately. Cluster analysis of EBV genes and host genes in patient NPC samples were performed. For each gene in all of the samples, the RPKM of all genes was added to 1 and then divided by the average gene expression across the samples. We then log-transformed the fold changes in gene expression and created gene expression heatmaps using the R language toolkit. The normalized expression data is clustered by the hierarchical clustering method.

### Statistical analysis

Statistical analyses were performed by two-tailed Student’s t-test using GraphPad prism 6.0 to determine the statistical significance between the experimental and control groups. P<0.05 was considered statistically significant. * P<0.05, ** P<0.01 and *** P<0.001; **** P<0.001; NS, not significant (P>0.05). Data were graphed as mean ±SEM.

## Supporting information

S1 Fig Establish HK-1 cell line in EBV abortive lytic phase. **(A)** The switch between EBV phases in EBV+ HK-1-Day7 and -Day14 was presented by GFP flow cytometry. **(B)** ZTA expression levels in EBV+ HK-1-Day7 and -Day14 were analyzed by Western blotting. **(C)** EBER1 expression levels were quantified by RT-PCR.
(PDF)

S2 Fig EBV lytic gene expression in EBV infected D7 and D14. **(A)** and **(B)** ZTA and EBER1 relative quantitative expression of Nasopharyngeal carcinoma cell lines in the different time points after EBV infection. **(C)** Heatmap illustrating relative EBV gene expression profiles in CNE2-EBV infected D7 and D14. Unsupervised clustering of genes (y-axis) and CNE2 in different EBV stage(x-axis) was performed by complete-linkage clustering.
(PDF)

S3 Fig CNE2 in EBV infected D7 has a stronger recruitment effect on THP-1. **(A)** Effect of CNE2 on recruiting THP-1 by Transwell migration assay (n = 3 per sample). Data are presented as the mean±SEM; * P < 0.*05*, ** P < 0.*01*, *** P < 0.*001*, **** P < 0.*0001*, *NS*, *not significant*.
(PDF)

S4 Fig HK-1 cell line in EBV abortive lytic phase exhibits the ability to recruit monocytes and induce their directed differentiations. **(A)** The number of each immune cells recruited by HK-1 in different EBV phase (n = 4). **(B)** The trend of cytokine mRNA level change was detected by real-time PCR (n = 3). **(C)** and **(D)** Supernatants of HK-1 with different EBV life cycle were used to treat DCs. The difference in the yield of DCs was examined by CD14&CD1a expression profile. The difference in the proportion of Mφs was examined by CD14&CD68 expression profile (n = 4). **(E)** Supernatants of HK-1 in different EBV life cycle were used to treat Mφs, the proportion of M1 and M2 subtypes were determined by detecting CD86 and CD163 (n = 4). Data are presented as the mean±SEM; * P < 0.*05*, ** P < 0.*01*, *** P < 0.*001*, **** P < 0.*0001*, *NS*, *not significant*.
(PDF)

S5 Fig ZTA exhibits the ability to recruit and induce directed differentiation of monocytes in HK-1. **(A)** The mRNA expression levels of viral genes were detected by real time PCR (n = 3). **(B)** Stable expression of ZTA in HK-1 cells confirmed by Western blot. **(C)** The mRNA expression levels of GM-CSF, IL-8, GRO-α were detected by real time PCR (n = 3). **(D)** The number of each immune cells recruited by HK-1-ZTA and HK-1-CTR (n = 4). **(E)** and **(F)** The change in the yield of DCs upon the treatment with the supernatants of HK-1-ZTA and HK-1-CTR was examined by CD14 and CD1a expression profile. The proportion of DC-d-Ms was examined by CD14 and CD68 (n = 4). **(G)** The proportion of M1 and M2 subtypes were determined by detecting CD86&CD163 (n = 4). Data are presented as the mean±SEM; * P < 0.*05*, ** P < 0.*01*, *** P < 0.*001*, **** P < 0.*0001*, *NS*, *not significant*.
(PDF)

S6 Fig ZTA-Knockout EBV+HK-1 cells lost the capacity of recruiting and polarizing monocyte. **(A)** The GFP profiles of EBV+ HK-1 after ZTA deletion were analyzed by flow cytometry. **(B)** The CRISPR/Cas9-mediated knockout efficiency of ZTA was estimated by measuring ZTA protein expression (n = 3). **(C)** EBER1 expression levels in the knockout and control cells were quantified by RT-PCR. **(D)** and **(E)** ZTA and EBER1 relative quantitative expression of Nasopharyngeal carcinoma cell lines in the different time points after ZTA was knocked out. **(F)** The mRNA expression levels of GM-CSF, IL-8, GRO-α in EBV+ HK-1 in ZTA-knockout and control cells were determined by real-time PCR (n = 3). **(G)** The number of each immune cells recruited by HK-1-EBV^+^SgCTR and HK-1-EBV^+^SgZTA (n = 4). **(H)** and **(I)** The yield of DCs upon the treatment with the supernatants of HK-1-EBV+ SgCTR and HK-1-EBV+ SgZTA was examined by CD14 and CD1a expression profile. The proportion of DC-d-Ms was examined by CD14 and CD68 (n = 4). **(J)** M1 and M2 proportions were identified by CD86 and CD163 expression (n = 4). Data are presented as the mean±SEM; * P < 0.*05*, ** P < 0.*01*, *** P < 0.*001*, **** P < 0.*0001*, *NS*, *not significant*.
(PDF)

S7 Fig EBV-DNA load was positively correlated with TAM and vessel density. **(A)** and **(B)**Spearman Rank Correlation Test was used to explore the correlation between EBV-DNA load and TAM/vessel density. * P < 0.*05*, ** P < 0.*01*, *** P < 0.*001*, **** P < 0.*0001*, *NS*, *not significant*.
(PDF)

S8 Fig EBV life cycle in HK-1 xenograft tumors was analyzed by immunohistochemical staining. **(A)** and **(B)** Representative images of HK-1 xenograft with different EBV phase, showing ZTA and EBNA1 expression. Cells were immunostained with the corresponding antibodies (400X). **(C)** The percentage of immunostaining positive cells in randomly chosen fields was analyzed using Image J (400X, n = 3 per sample). **(D)** and **(E)** Representative images of HK-1-CTR and HK-1-ZTA xenograft, showing ZTA expression(400X). The percentage of ZTA positive cells in randomly chosen fields was quantified using Image J (n = 3 per sample). **(F)** and **(G)**, Representative images of HK-1-EBV+ SgCTR and HK-1-EBV+ SgZTA xenograft, showing ZTA and EBNA1 expression. The percentage of immunostaining positive cells in randomly chosen fields was quantified using Image J (n = 3 per sample). Data are presented as the mean±SEM; * P < 0.*05*, ** P < 0.*01*, *** P < 0.*001*, **** P < 0.*0001*, *NS*, *not significant*.
(PDF)

S9 Fig CCL5 was not regulated by EBV abortive lytic cycle. **(A)** the qPCR analysis and ct value of CCL5 in Fig 2F. **(B)** CCL5 expression was down-regulated in advanced clinical samples. * P < 0.*05*, ** P < 0.*01*, *** P < 0.*001*, **** P < 0.*0001*, *NS*, *not significant*.
(PDF)

S1 Table PCR primer list of EBV lytic genes. The primer sequence were designed by PRIMER PREMIE5 and were validated by sequencing the final constructed plasmid. S1 Table was uploaded separately as an excel file.
(XLSX)

S2 Table Primer list for real time PCR. For each target gene, we designed primer sequences from PRIMER BANK, and the amplified fragments were about 100bp. S2 Table was uploaded separately as an excel file.
(XLSX)

S3 Table The RPKM value of host genes in RNA-Seq data. This portion of RNA-Seq data were donated by Dr. Mu-Sheng Zeng at Sun Yat-sen University Cancer Center. Raw reads from RNA-Seq data were uniformly processed and converted to RPKM values.
(XLSX)

S4 Table The RPKM value of EBV genes in RNA-Seq data. This portion of RNA-Seq data were donated by Dr. Mu-Sheng Zeng at Sun Yat-sen University Cancer Center. Raw reads from RNA-Seq data were uniformly processed and converted to RPKM values.
(XLSX)
