# Supplementary figures and images for: EBV abortive lytic cycle promotes nasopharyngeal carcinoma progression through recruiting monocytes and regulating their directed differentiation
Source: PLoS Pathog. 2024 Jan 11;20(1):e1011934. doi: 10.1371/journal.ppat.1011934 (PMC10846743; doi:10.1371/journal.ppat.1011934)

Supplemental Fig. S1

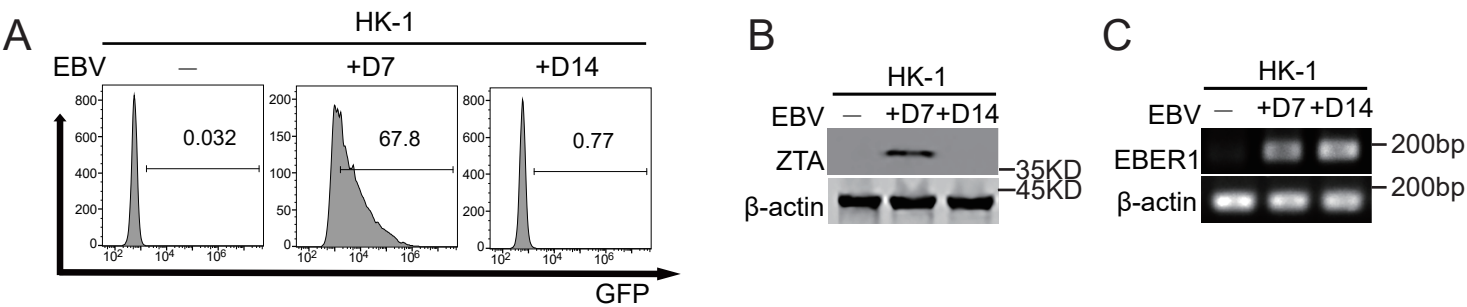

Supplement: S1 Fig — (A) The switch between EBV phases in EBV+ HK-1-Day7 and -Day14 was presented by GFP flow cytometry. (B) ZTA expression levels in EBV+ HK-1-Day7 and -Day14 were analyzed by Western blotting. (C) EBER1 expression levels were quantified by RT-PCR. (PDF) [file ppat.1011934.s001.pdf]

## Supplemental Fig. S2

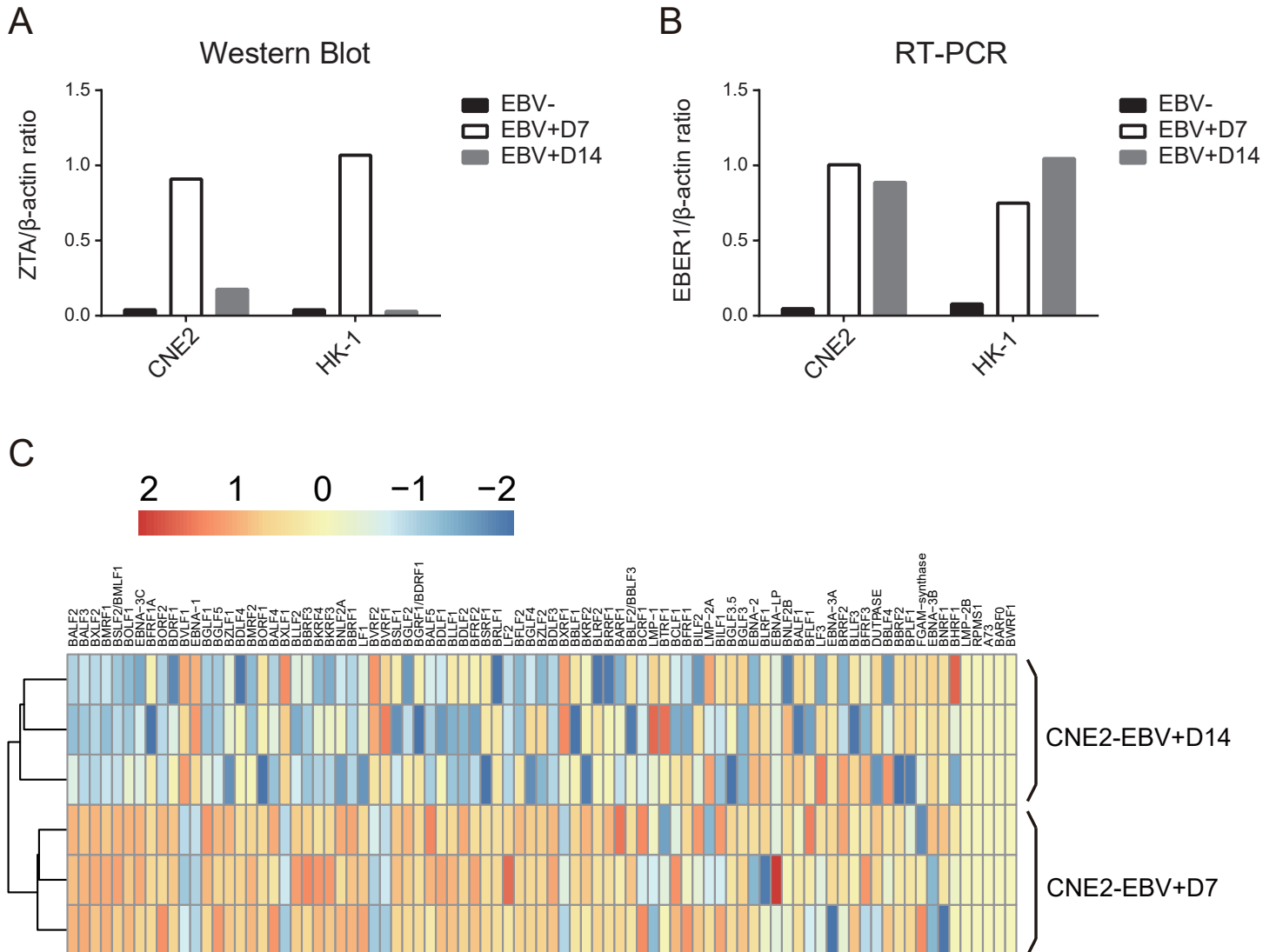

Supplement: S2 Fig — (A) and (B) ZTA and EBER1 relative quantitative expression of Nasopharyngeal carcinoma cell lines in the different time points after EBV infection. (C) Heatmap illustrating relative EBV gene expression profiles in CNE2-EBV infected D7 and D14. Unsupervised clustering of genes (y-axis) and CNE2 in different EBV stage(x-axis) was performed by complete-linkage clustering. (PDF) [file ppat.1011934.s002.pdf]

Supplemental Fig. S3

A

THP-1  
migration

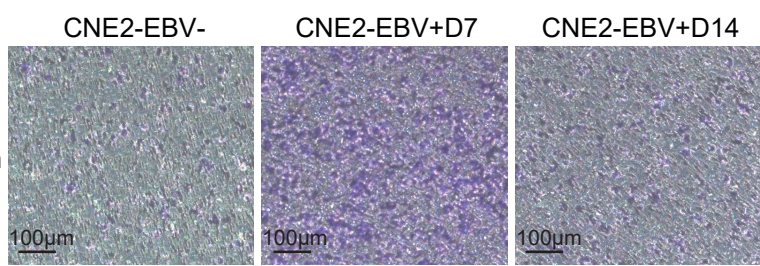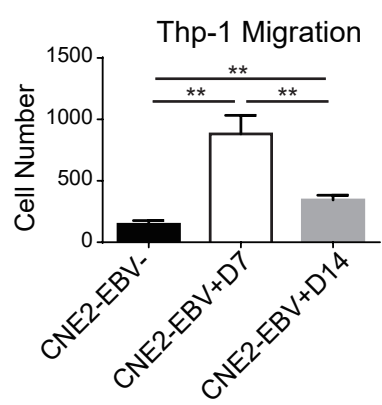

Supplement: S3 Fig — (A) Effect of CNE2 on recruiting THP-1 by Transwell migration assay (n = 3 per sample). Data are presented as the mean±SEM; * P < 0.05, ** P < 0.01, *** P < 0.001, **** P < 0.0001, NS, not significant. (PDF) [file ppat.1011934.s003.pdf]

Supplemental Fig. S4

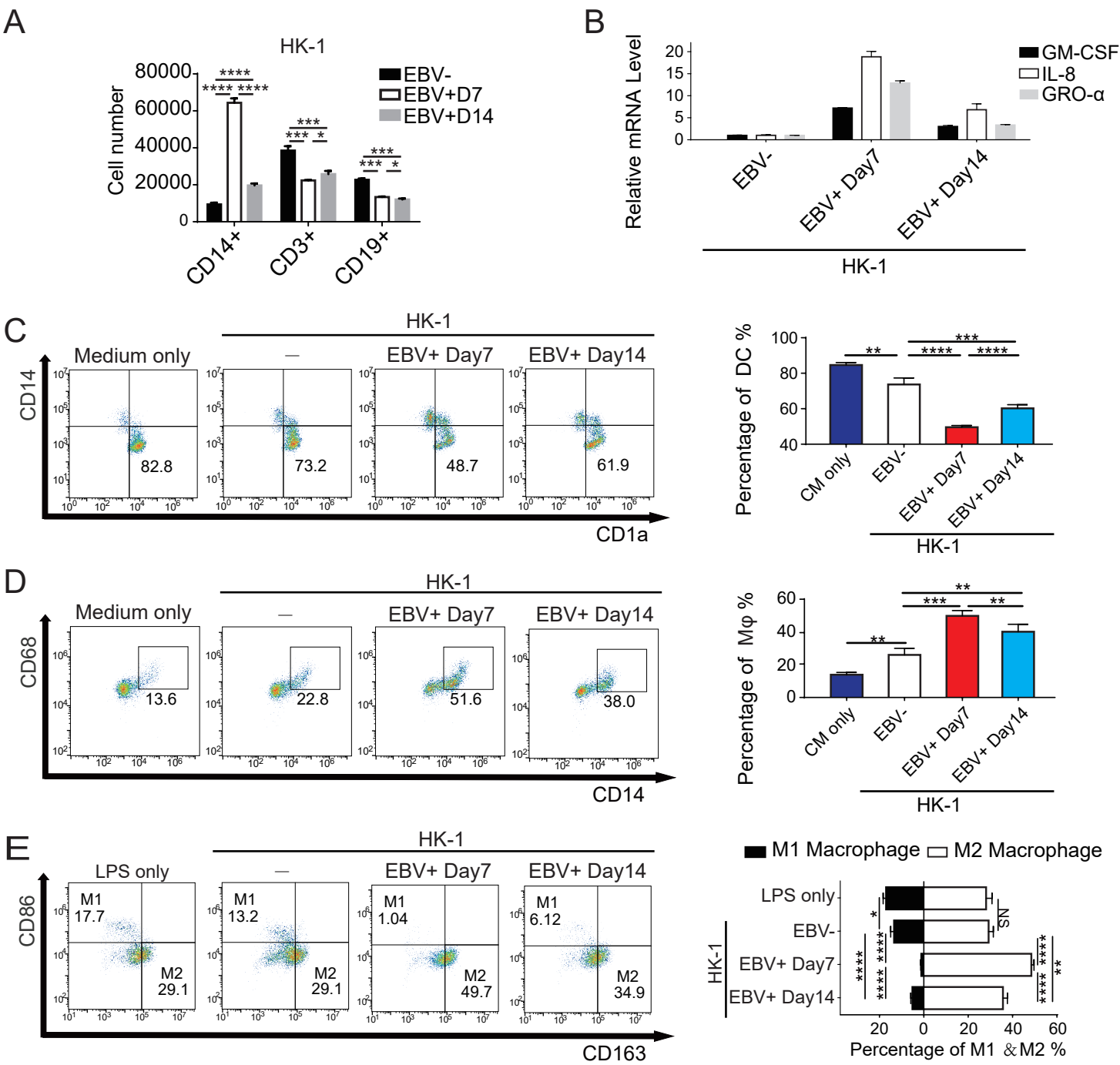

Supplement: S4 Fig — (A) The number of each immune cells recruited by HK-1 in different EBV phase (n = 4). (B) The trend of cytokine mRNA level change was detected by real-time PCR (n = 3). (C) and (D) Supernatants of HK-1 with different EBV life cycle were used to treat DCs. The difference in the yield of DCs was examined by CD14&CD1a expression profile. The difference in the proportion of Mφs was examined by CD14&CD68 expression profile (n = 4). (E) Supernatants of HK-1 in different EBV life cycle were used to treat Mφs, the proportion of M1 and M2 subtypes were determined by detecting CD86 and CD163 (n = 4). Data are presented as the mean±SEM; * P < 0.05, ** P < 0.01, *** P < 0.001, **** P < 0.0001, NS, not significant. (PDF) [file ppat.1011934.s004.pdf]

Supplemental Fig. S5

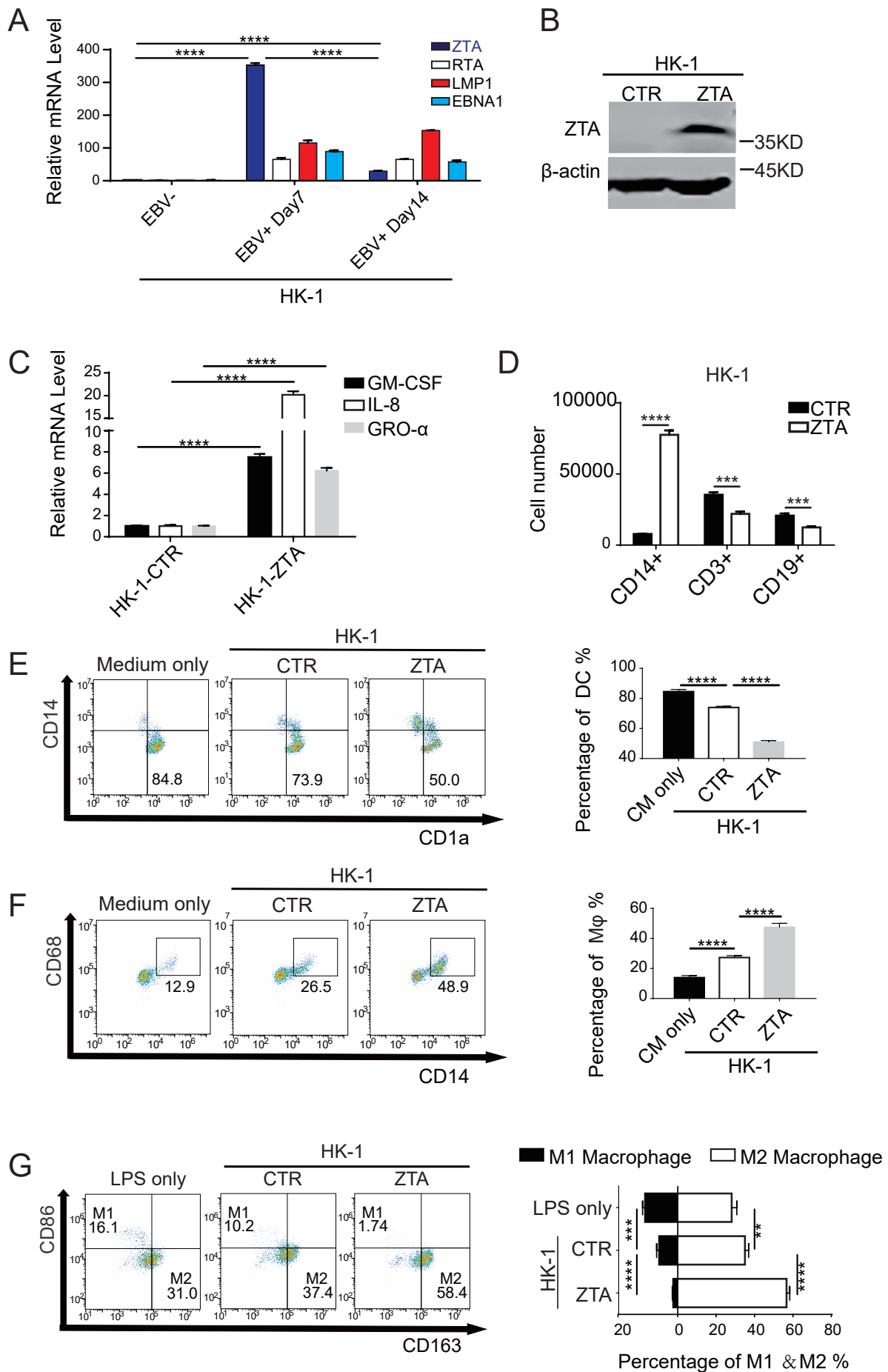

Supplement: S5 Fig — (A) The mRNA expression levels of viral genes were detected by real time PCR (n = 3). (B) Stable expression of ZTA in HK-1 cells confirmed by Western blot. (C) The mRNA expression levels of GM-CSF, IL-8, GRO-α were detected by real time PCR (n = 3). (D) The number of each immune cells recruited by HK-1-ZTA and HK-1-CTR (n = 4). (E) and (F) The change in the yield of DCs upon the treatment with the supernatants of HK-1-ZTA and HK-1-CTR was examined by CD14 and CD1a expression profile. The proportion of DC-d-Ms was examined by CD14 and CD68 (n = 4). (G) The proportion of M1 and M2 subtypes were determined by detecting CD86&CD163 (n = 4). Data are presented as the mean±SEM; * P < 0.05, ** P < 0.01, *** P < 0.001, **** P < 0.0001, NS, not significant. (PDF) [file ppat.1011934.s005.pdf]

# Supplemental Fig. S6

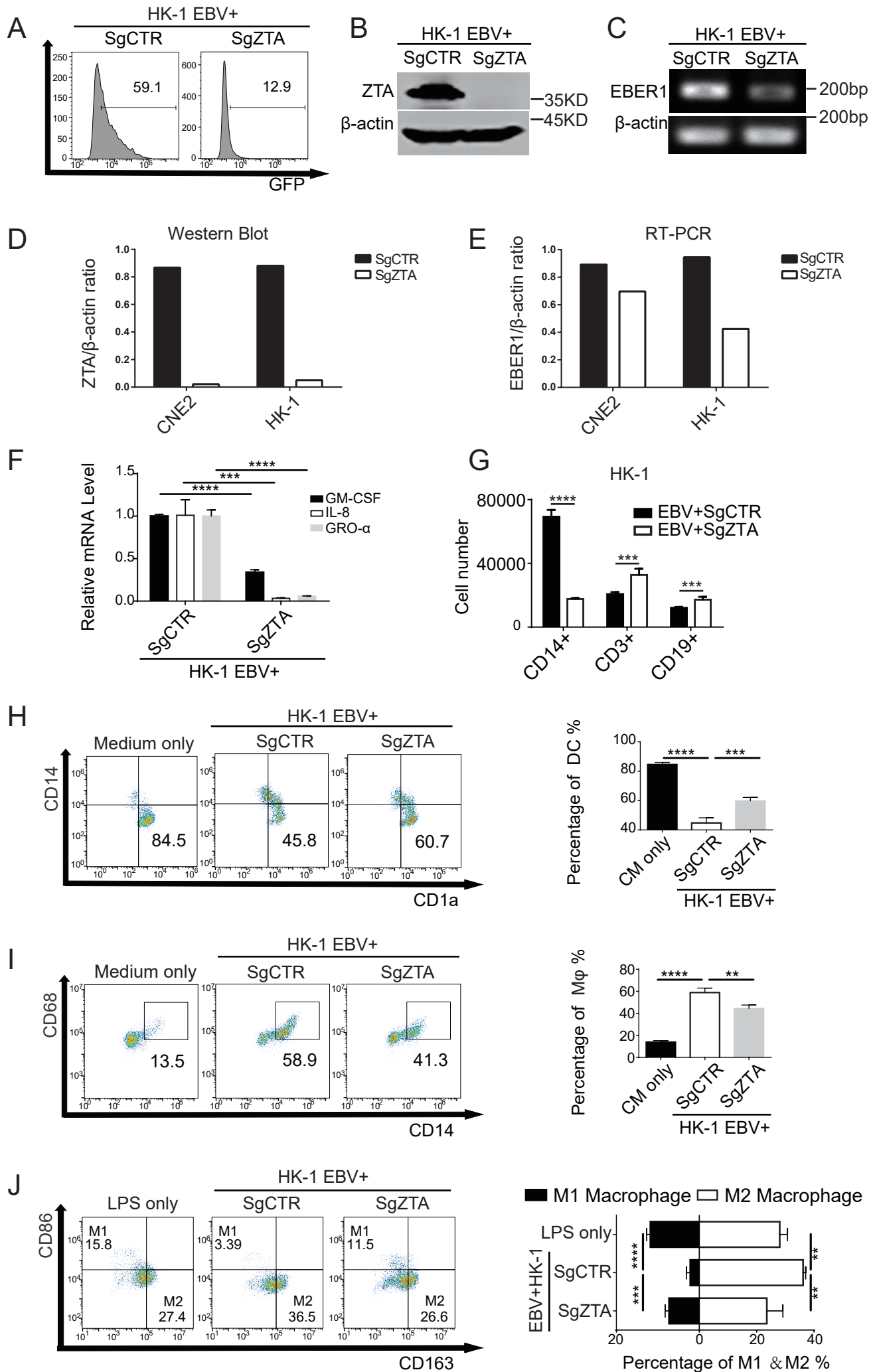

Supplement: S6 Fig — (A) The GFP profiles of EBV+ HK-1 after ZTA deletion were analyzed by flow cytometry. (B) The CRISPR/Cas9-mediated knockout efficiency of ZTA was estimated by measuring ZTA protein expression (n = 3). (C) EBER1 expression levels in the knockout and control cells were quantified by RT-PCR. (D) and (E) ZTA and EBER1 relative quantitative expression of Nasopharyngeal carcinoma cell lines in the different time points after ZTA was knocked out. (F) The mRNA expression levels of GM-CSF, IL-8, GRO-α in EBV+ HK-1 in ZTA-knockout and control cells were determined by real-time PCR (n = 3). (G) The number of each immune cells recruited by HK-1-EBV+SgCTR and HK-1-EBV+SgZTA (n = 4). (H) and (I) The yield of DCs upon the treatment with the supernatants of HK-1-EBV+ SgCTR and HK-1-EBV+ SgZTA was examined by CD14 and CD1a expression profile. The proportion of DC-d-Ms was examined by CD14 and CD68 (n = 4). (J) M1 and M2 proportions were identified by CD86 and CD163 expression (n = 4). Data are presented as the mean±SEM; * P < 0.05, ** P < 0.01, *** P < 0.001, **** P < 0.0001, NS, not significant. (PDF) [file ppat.1011934.s006.pdf]

Supplemental Fig. S8

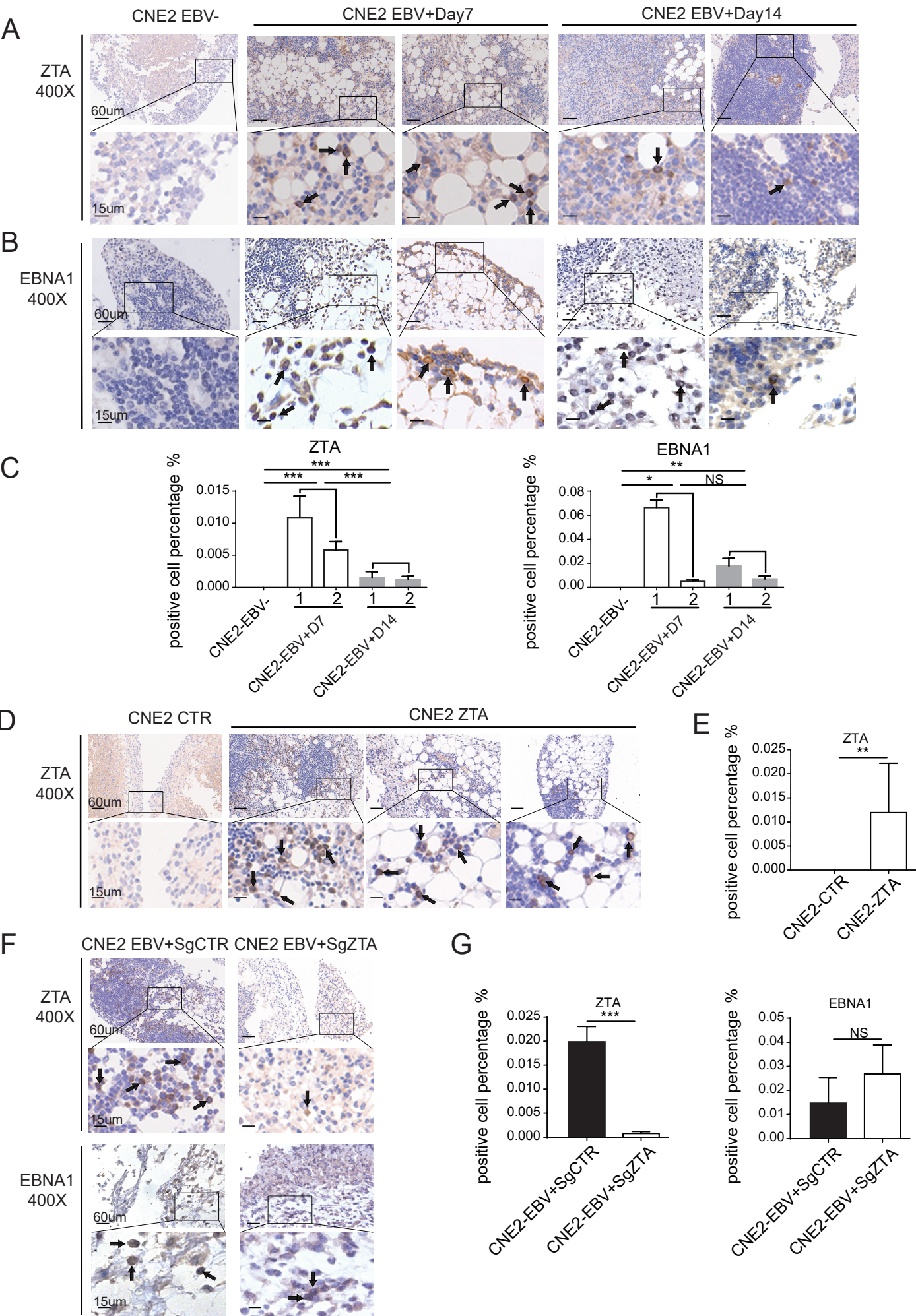

Supplement: S8 Fig — (A) and (B) Representative images of HK-1 xenograft with different EBV phase, showing ZTA and EBNA1 expression. Cells were immunostained with the corresponding antibodies (400X). (C) The percentage of immunostaining positive cells in randomly chosen fields was analyzed using Image J (400X, n = 3 per sample). (D) and (E) Representative images of HK-1-CTR and HK-1-ZTA xenograft, showing ZTA expression(400X). The percentage of ZTA positive cells in randomly chosen fields was quantified using Image J (n = 3 per sample). (F) and (G), Representative images of HK-1-EBV+ SgCTR and HK-1-EBV+ SgZTA xenograft, showing ZTA and EBNA1 expression. The percentage of immunostaining positive cells in randomly chosen fields was quantified using Image J (n = 3 per sample). Data are presented as the mean±SEM; * P < 0.05, ** P < 0.01, *** P < 0.001, **** P < 0.0001, NS, not significant. (PDF) [file ppat.1011934.s008.pdf]

Supplemental Fig. S9

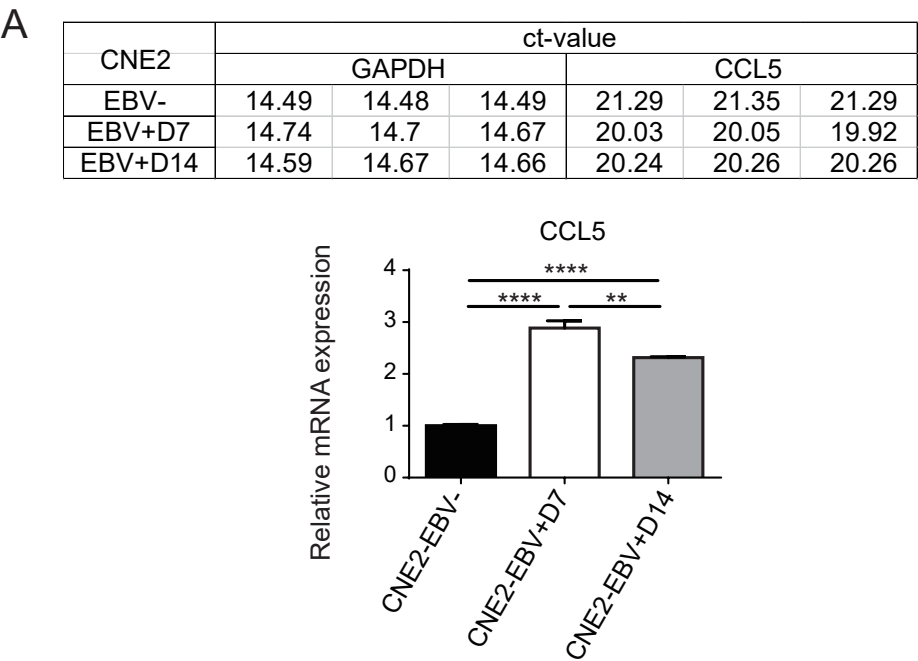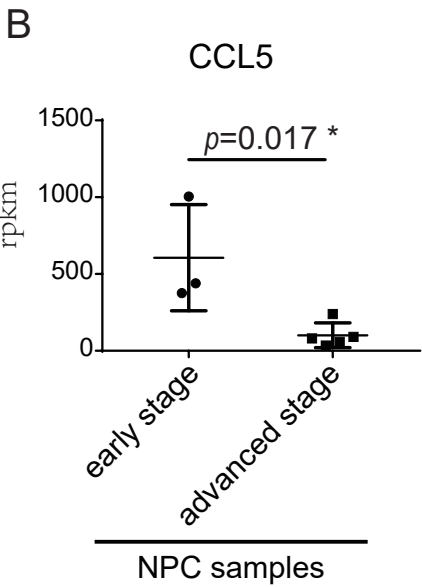

Supplement: S9 Fig — (A) the qPCR analysis and ct value of CCL5 in Fig 2F. (B) CCL5 expression was down-regulated in advanced clinical samples. * P < 0.05, ** P < 0.01, *** P < 0.001, **** P < 0.0001, NS, not significant. (PDF) [file ppat.1011934.s009.pdf]
