# Supplementary material for: EBV abortive lytic cycle promotes nasopharyngeal carcinoma progression through recruiting monocytes and regulating their directed differentiation
Source: PLoS Pathog. 2024 Jan 11;20(1):e1011934. doi: 10.1371/journal.ppat.1011934 (PMC10846743; doi:10.1371/journal.ppat.1011934)

Supplemental Fig. S7

A

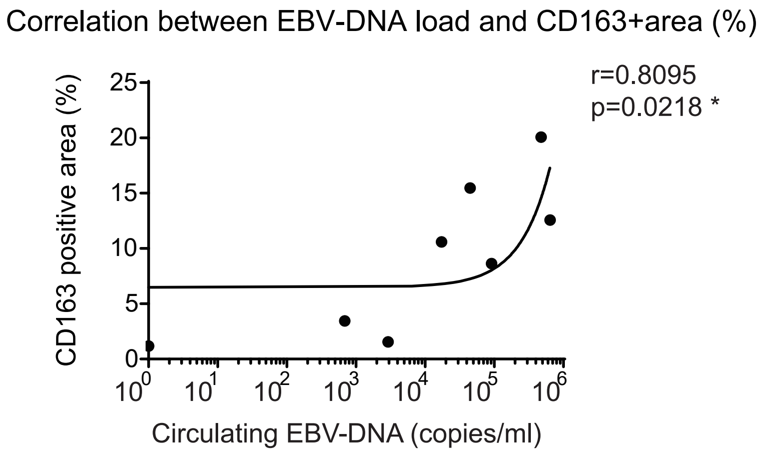

B

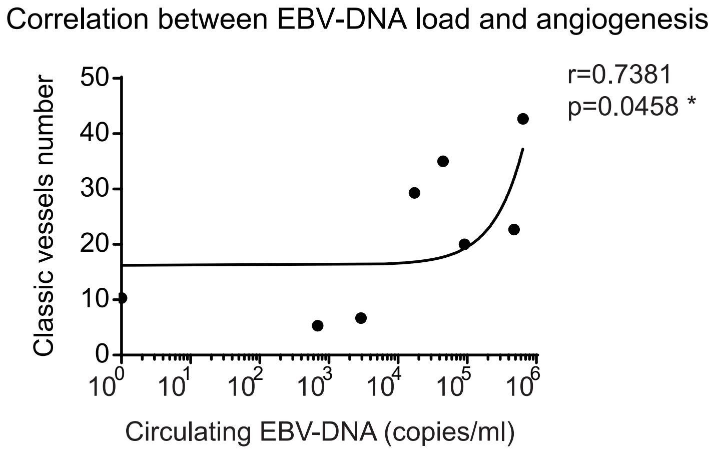

Supplement: S7 Fig — (A) and (B)Spearman Rank Correlation Test was used to explore the correlation between EBV-DNA load and TAM/vessel density. * P < 0.05, ** P < 0.01, *** P < 0.001, **** P < 0.0001, NS, not significant. (PDF) [file ppat.1011934.s007.pdf]
